# Mechanism of NanR transcriptional activation of sialic acid metabolism in *Streptococcus pneumoniae*

**DOI:** 10.1093/nar/gkag953

**Published:** 2026-10-05

**Authors:** David M Wood, Ashleigh S Johns, Zachary D Tillett, Heather L Shearer, Santosh Panjikar, Yee-Foong Mok, Michael D W Griffin, Timothy M Allison, Rachel A North, Paul E Pace, Borries Demeler, Mark B Hampton, Renwick C J Dobson, Christopher R Horne

**Affiliations:** Biomolecular Interaction Centre and School of Biological Sciences, University of Canterbury, PO Box 4800, Christchurch 8140, New Zealand; Biomolecular Interaction Centre and School of Biological Sciences, University of Canterbury, PO Box 4800, Christchurch 8140, New Zealand; Biomolecular Interaction Centre and School of Biological Sciences, University of Canterbury, PO Box 4800, Christchurch 8140, New Zealand; Mātai Hāora—Centre for Redox Biology and Medicine, Department of Pathology and Biomedical Science, University of Otago Christchurch, Christchurch 8041, New Zealand; Australian Synchrotron, ANSTO, Clayton, 800 Blackburn Road, Victoria 3168, Australia; Department of Biochemistry and Molecular Biology, Monash University, Melbourne 3800 VIC, Australia; Department of Biochemistry and Pharmacology, Bio21 Molecular Science and Biotechnology Institute, University of Melbourne, Parkville, Victoria 3010, Australia; Department of Biochemistry and Pharmacology, Bio21 Molecular Science and Biotechnology Institute, University of Melbourne, Parkville, Victoria 3010, Australia; Biomolecular Interaction Centre, School of Physical and Chemical Sciences, University of Canterbury, Christchurch 8041, New Zealand; School of Medical Sciences, Faculty of Medicine and Health, University of Sydney, New South Wales 2006, Australia; Mātai Hāora—Centre for Redox Biology and Medicine, Department of Pathology and Biomedical Science, University of Otago Christchurch, Christchurch 8041, New Zealand; Department of Chemistry and Biochemistry, University of Lethbridge, 4401 University Drive, Lethbridge, AB T1K 3M4, Canada; Department of Chemistry and Biochemistry, University of Montana, Missoula, MT 59812, United States; Mātai Hāora—Centre for Redox Biology and Medicine, Department of Pathology and Biomedical Science, University of Otago Christchurch, Christchurch 8041, New Zealand; Biomolecular Interaction Centre and School of Biological Sciences, University of Canterbury, PO Box 4800, Christchurch 8140, New Zealand; Department of Biochemistry and Pharmacology, Bio21 Molecular Science and Biotechnology Institute, University of Melbourne, Parkville, Victoria 3010, Australia; Biomolecular Interaction Centre and School of Biological Sciences, University of Canterbury, PO Box 4800, Christchurch 8140, New Zealand; Walter and Eliza Hall Institute of Medical Research, 1G Royal Parade, Parkville, VIC 3050, Australia; Department of Medical Biology, University of Melbourne, Parkville, Victoria 3052, Australia; Drug Discovery Biology, Monash Institute of Pharmaceutical Sciences, Monash University, Victoria 3052, Australia

## Abstract

In *Streptococcus pneumoniae*, the RpiR transcriptional regulator NanR (*Sp*NanR) senses sialic acid in the environment and upregulates transcription of the *nan* and *sia* A operons to increase uptake and metabolism of sialic acid. The molecular basis of this activation is unknown. Here, we demonstrate that *Sp*NanR binds *N*-acetylmannosamine-6-phosphate, a metabolite of sialic acid catabolism. *Sp*NanR exists in a dimer–tetramer equilibrium, and *N*-acetylmannosamine-6-phosphate binding strongly stabilizes the tetramer. Crystal structures and site-specific substitutions demonstrate that *N*-acetylmannosamine-6-phosphate bridges and stabilizes the *Sp*NanR tetramer. *Sp*NanR binds its DNA recognition sequence with nanomolar affinity. Notably, the effector *N*-acetylmannosamine-6-phosphate does not affect the affinity of *Sp*NanR for DNA. The DNA-binding domains are not structurally coupled to the sugar isomerase domains, explaining why *N*-acetylmannosamine-6-phosphate binding does not affect DNA binding. Structural analysis reveals that sequence specificity arises through distortion of B-DNA and an unusual π-stack formed by two arginine residues in the minor groove, while affinity is driven by backbone contacts. We propose a mechanism by which *S. pneumoniae* regulates sialic acid metabolism, consistent with our biophysical experiments and *in vivo* regulatory behavior. These findings define a unique activation mechanism for an RpiR regulator and provide new insights into carbohydrate-responsive gene regulation in pneumococci.

## Graphical Abstract

**Graphical Abstract:**
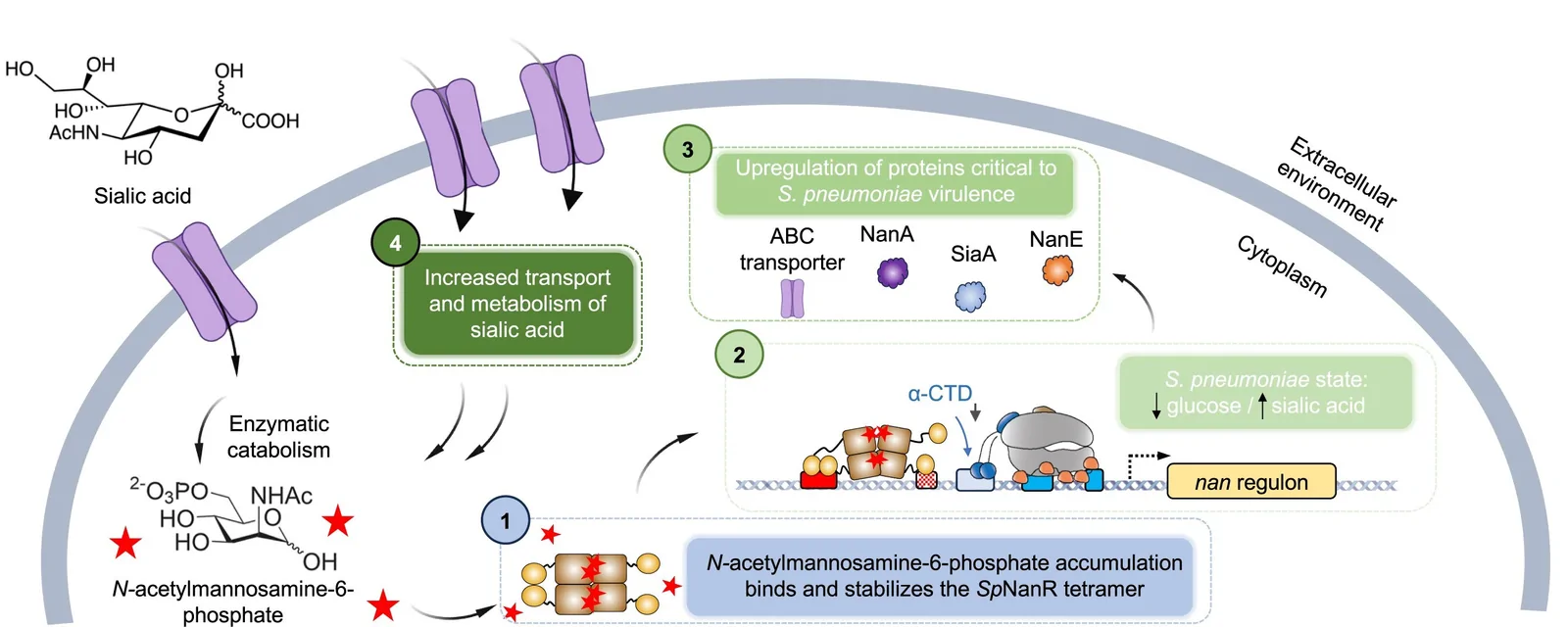

## Introduction

Transcriptional gene regulation enables a cell to tailor its gene expression in response to a molecular signal. For example, when a cell detects changes in the environmental nutrient levels, genes encoding the appropriate machinery are induced, while genes that are not required are repressed. In this way, bacteria optimize growth and improve their fitness within a new environment by altering transcription. However, the molecular mechanisms that govern such decisions are often unknown. Here, we define how a ribose phosphate isomerase regulator (RpiR) protein controls the expression of genes required for the uptake and utilization of an alternative carbohydrate in *Streptococcus pneumoniae*.

The ability to metabolize alternative carbohydrates, such as sialic acids, is a known virulence factor for bacterial colonization and pathogenesis [1–4]. Sialic acids are amino monosaccharides commonly found conjugated to the surface of mammalian cells [5], where they serve as markers of “self” for the human immune system [6, 7]. Some bacteria, including *S. pneumoniae*, cleave, import, and metabolize sialic acids derived from mammalian glycoconjugates [8, 9], reviewed in North *et al*., Dhanabalan *et al*., and Kim *et al*. [10–12]. Disrupting the genes responsible for sialic acid import [4, 13, 14] or catabolism [15–17] decreases microbial pathogenesis. Bacteria import sialic acids for several reasons: to provide precursors (mannosamine and glucosamine) for cell wall synthesis [18]; to enable catabolism via glycolysis for energy production and serve as a source of carbon and nitrogen precursors [19]; and, in some species, to allow repurposed sialic acids to be displayed on their own cell surface, facilitating immune evasion through molecular mimicry [7, 20, 21].

The ability to metabolize sialic acids by *S. pneumoniae* is conferred by the *nan* gene cluster [22, 23]. While it varies slightly amongst pneumococcal strains, the *nan* gene cluster typically comprises two *nan* operons and a *sia*A operon (Fig. 1A). *Nan* operon-I encodes catabolic enzymes that convert sialic acid to *N-*acetylglucosamine-6-phosphate through a series of steps [24–28], while *nan* operon-II encodes processing enzymes and an ABC transporter. The *sia*A operon encodes a sialidase responsible for cleaving extracellular, host-derived sialic acids conjugated on mammalian cell surfaces [23].

**Figure 1.**
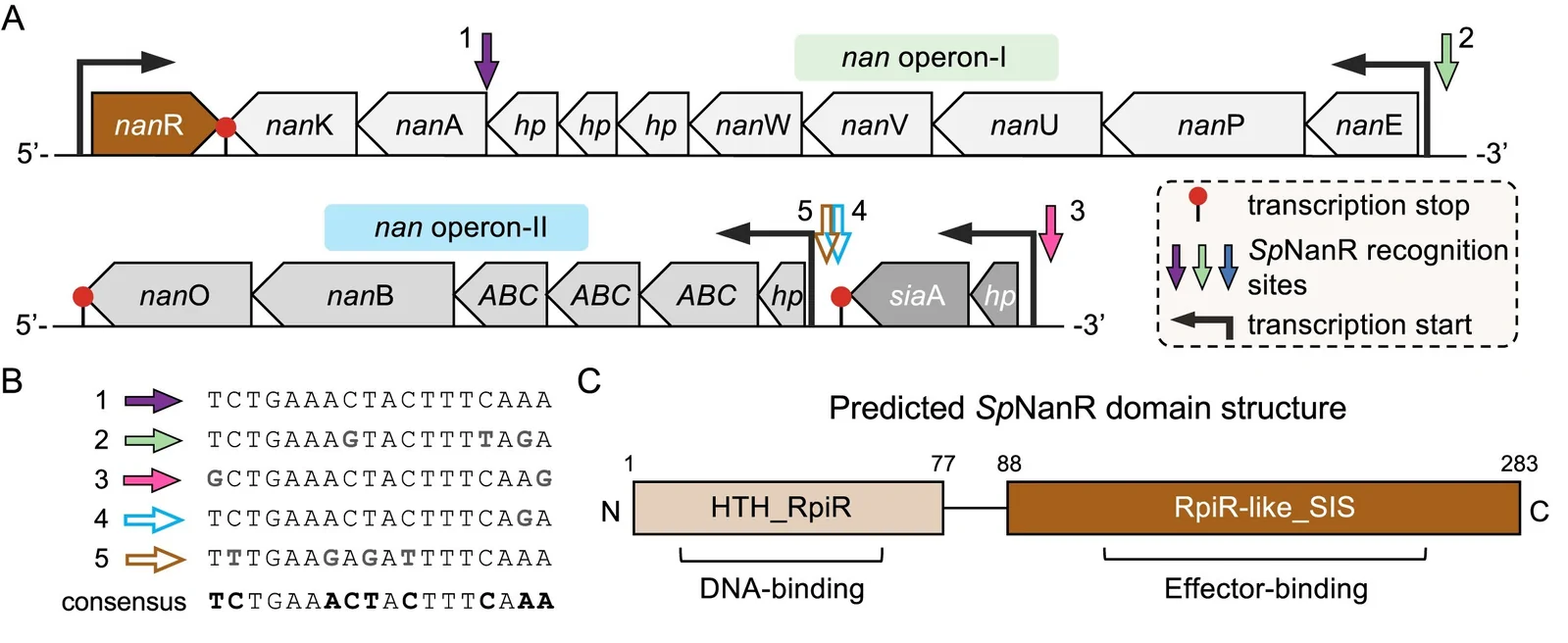
Organization of the *nan* gene cluster and *Sp*NanR recognition sequences. (**A**) The *nan* cluster transcripts show the location of each gene, including *nan*R (*Sp*NanR, brown). Black arrows are located at transcription start sites and red lollipops denote transcription stop sites. Solid purple, green, and pink arrows show the location of the *Sp*NanR recognition sequences annotated from strain D39, NC_008533.2. Nan operon-I (in the direction of transcription): *nan*E *(N*-acetylmannosamine-6-phosphate 2-epimerase), *nan*P (PTS, IIBC components), *nan*U (ABC sugar-binding protein), *nan*V (ABC transporter permease), *nan*W (ABC carbohydrate transporter), *hp* (hypothetical proteins), *nan*A (*N*-acetylneuraminate lyase), and *nan*K (*N*-acetylneuraminate kinase). Nan operon-II: *hp* (hypothetical protein), *ABC* (ABC substrate-binding protein, transporter permease, carbohydrate transporter), *nan*B (neuramidase), and *nan*O (oxidoreductase). *sia*A operon: *hp* (hypothetical protein) and *sia*A (sialidase, sometimes annotated as *nan*A). Putative recognition sites are annotated by open blue and brown arrows (see results). (**B**) The *Sp*NanR recognition sequences vary slightly and the consensus sequence is 5′TCTGAAACTACTTTCAAA3′. The five locations that form the consensus sequence in the *nan* gene cluster are marked by numbered arrows in panel (A), and the sequences are detailed here. Variations in the base pairs are in bold. (**C**) Domain structure of *Sp*NanR, as predicted by InterPro [45]. HTH refers to a helix–turn–helix domain, while SIS refers to a sugar isomerase domain.

The proteins *Sp*NanR and the catabolite control protein A (CcpA) regulate transcription of the genes that allow for sialic acid utilization in *S. pneumoniae* [22, 23, 29]. *Sp*NanR is predicted to recognize an 18 base pair near palindromic DNA sequence, which has been identified at three sites within the *nan* gene cluster (Fig. 1A and B, solid arrows). We subsequently identified two further putative sites (Fig. 1A and B, open arrows), which are discussed later. In the presence of sialic acid but absence of glucose, expression of *nan* operon-I and the *sia*A operon is significantly increased; this effect is abolished upon deletion of *Sp*NanR [22] indicating that *Sp*NanR functions as a transcriptional activator. However, in the presence of glucose and sialic acid, transcription of these operons is not induced. Repression is attributed to the phosphorylation of the co-repressor HPr, which becomes phosphorylated under high-glucose conditions and subsequently binds to CcpA. CcpA recognizes a catabolite repression element (cre), a pseudo-palindromic consensus sequence (5′tTGAAAGtGtTTaCAa3′) [29]. Binding of CcpA to the −35 region sterically occludes RNA polymerase recruitment, and the consensus sequence is present within the promoter regions of the *nan* operon-I and the *sia*A operon. *Streptococcus pneumoniae* displays classical diauxic growth in mixed-carbon environments, reflecting hierarchical regulation of carbon utilization mediated by CcpA. Collectively, these observations support a model in which sialic acid serves as a secondary carbon source, with *Sp*NanR-dependent transcription overridden under glucose-replete conditions [30].

*Sp*NanR is an RpiR transcriptional regulator comprising two domains (Fig. 1C). The N-terminal DNA-binding domain is a well-conserved helix–turn–helix motif, defined by a tri-helical structure where the third α-helix is often referred to as the “recognition helix” and interacts directly with the DNA major groove [31]. The C-terminal domain is a sugar isomerase domain, a common feature in regulators of sugar-phosphate metabolism [32]. *N*-Acetylmannosamine or *N*-acetylmannosamine-6-phosphate, which are both sialic acid derivatives, is proposed to modulate transcription of the *nan* gene cluster, presumably through binding the *Sp*NanR sugar isomerase domain [22, 23]. *N*-Acetylmannosamine-6-phosphate is an important intermediate in bacterial sialic acid catabolism which, through the epimerase NanE, can increase levels of *N-*acetylglucosamine-6-phosphate. This metabolite can be converted to fructose-6-phosphate, a central intermediate in carbon metabolism by the deacetylase NagA and deaminase NagB enzymes [26, 33], or fed into peptidoglycan synthesis via UDP-GlcNAc, an essential substrate for building the peptidoglycan layer of the cell wall [34].

piR transcription regulators control a variety of processes within bacteria [35–39], but are understudied at the mechanistic and structural level. Five independent domain structures have been determined experimentally: two for the N-terminal DNA-binding domain (PDB ID: 3IWF; 203F, both unpublished) and three for the sugar isomerase domain [(PDB ID: 3SHO, unpublished; 3CVJ, unpublished; 7EN6 [40]). In addition, there are two full-length RpiR structures: NanR protein from *Vibrio vulnificus* (*Vv*NanR) in complex with *N*-acetylmannosamine-6-phosphate (PDB ID: 4IVN [41]) and the recently reported *Pseudomonas aeruginosa* RccR regulator of carbon metabolism (PDB ID: 8JU9 [42, 43]). To date, no crystal structures of full-length RpiR bound to DNA or in the apo form (i.e. without effector) have been reported, representing a key knowledge gap in understanding the molecular mechanism of RpiR transcriptional regulators.

Here, we report on the molecular mechanism of sialic acid regulation in *S. pneumoniae*. We identify the effector that binds *Sp*NanR to regulate expression of the *nan* and *sia*A operons. *N-*Acetylmannosamine-6-phosphate binding modulates the oligomeric state of *Sp*NanR but does not affect the affinity of *Sp*NanR for DNA. We report the first full-length structures of the RpiR family transcriptional regulator *Sp*NanR, including the unbound (apo) *Sp*NanR crystal structure, *Sp*NanR in complex with *N*-acetylmannosamine-6-phosphate, and *Sp*NanR in complex with its cognate DNA recognition sequence. Together, these results allow us to propose a molecular mechanism for the interplay between sialic acid, operon structure, and the transcriptional regulator *Sp*NanR, which affords a competitive advantage to the bacterium *S. pneumoniae* during host colonization.

## Materials and methods 

DNA oligonucleotides were synthesized and annealed by GenScript. Upon receipt they were resuspended in SEC buffer [20 mM Tris–HCl (pH 8.0), 300 mM NaCl] at 100 mM concentrations and stored at −20°C. DNA that was used in fluorescent experiments was labeled with 5′-FAM on the sense strand.

### Expression and purification of recombinant *Sp*NanR

The protein sequence corresponding to *S. pneumoniae* NanR (gene: SPN23F_RS08445 NCBI Reference Sequence: WP_000360349.1) was synthesized by GenScript and cloned into pET30ΔSE. Mutant sequences were ordered from GenScript directly.

The pET30ΔSE-*Sp*NanR and mutated constructs were transformed into *Escherichia coli* BL21(DE3) competent cells (Agilent) and grown in LB media with 30 mg.ml^−1^ kanamycin at 37°C and shaking at 220 rpm. Expression of *Sp*NanR was induced mid-log phase (OD_600_ ≈ 0.6) by the addition of Isopropyl β-D-1-thiogalactopyranoside (IPTG) to a final concentration of 1 mM and incubated overnight (14–16 h) at 25°C with shaking at 220 rpm. Cells were harvested by centrifugation (Sorvall LYNX 4000 Superspeed) at 8000 rpm for 10 min. Following centrifugation, the supernatant was discarded and the cell pellet was flash cooled in liquid nitrogen for storage until needed.

To prepare the protein for purification, the cell pellet was resuspended in lysis buffer (20 mM Tris–HCl, pH 8.0, 150 mM NaCl), supplemented with cOmplete™ mini protease cocktail inhibitor (Roche), and lysed by sonication (Hielscher UP200S Ultrasonic Processor) on ice. Cell debris and insoluble material was pelleted by centrifugation at 16 000 rpm for 30 min. The resulting cell lysate was then saturated to 50% ammonium sulphate and left to equilibrate for 1 h at 4°C. Following precipitation, protein was pelleted at 10 000 rpm for 15 min and resuspended in buffer A (20 mM Tris–HCl, pH 8.0, 100 mM NaCl), while the supernatant was discarded. This resuspended sample was dialyzed overnight in buffer A at 4°C.

Purification of *S. pneumoniae* NanR was conducted using a three-step procedure on an ÄKTApure (Cytiva): anion exchange chromatography, heparin affinity chromatography, and size-exclusion chromatography (Supplementary Fig. S1A). The dialyzed sample was applied to a HisTrap Q FF column (Cytiva), pre-equilibrated in buffer A. Weakly associated or positively charged proteins were removed by washing the column for three column volumes of buffer A. Subsequently, the bound protein was eluted using a continuous gradient of buffer B (20 mM Tris–HCl, pH 8.0, 1 M NaCl) over 60 min. Fractions containing protein were identif odium dodecyl sulfate–polyacrylamide gel electrophoresis and then pooled. The pooled sample was then applied to a Hi-Trap Heparin HP column (Cytiva), pre-equilibrated in buffer A. Next, the column was washed using three column volumes of buffer A, while the bound protein was eluted using a continuous gradient of buffer B over 20 min. Following concentration to >500 μl using ultrafiltration (Sartorius), the pooled sample was applied to a Superdex 200 Increase 10/300 GL column in SEC buffer (20 mM Tris–HCl, pH 8.0, 300 mM NaCl) where *Sp*NanR eluted as a single peak. The absorbance at 280 nm of the purified protein was measured using a NanoDrop spectrophotometer and the concentration was estimated using a molar extinction coefficient of 27 390 M^−1^.cm^−1^ at 280 nm as calculated by ProtParam [44]. All purification steps were carried out at 4°C. Protein that was not immediately used in experiments was flash-frozen in liquid nitrogen and stored at −80°C. We verified the mass of our protein using intact mass spectrometry, which shows that the protein has a mass of 32 611 Da (Supplementary Fig. S1B). Domain structure of *Sp*NanR was predicted using InterPro (45). 

### Differential scanning fluorimetry

A QuantStudio 3 real-time PCR system (ThermoFisher Scientific) was used to record the thermal stability of the purified protein with or without potential substrates. Reactions consisted of *Sp*NanR (0.65 mg.ml^−1^, 20 μM), 5× SYPRO® Orange (prepared as a 50× stock), and 1 mM effector in a final reaction volume of 25 μl. Once prepared these were loaded into a 96-well microplate (Applied Biosystems). Samples were heated from 4°C to 95°C at a ramp rate of 0.05°C, taking fluorescent readings at each time point. Triplicate measurements were performed for each sample. To validate the experiment three negative controls (protein only, dye only, ligand only) were conducted. Data analysis was performed using Protein Thermal Shift software (version 1.3-Applied Biosystems) where an apparent melting point (*T*_m_) of each sample in °C was obtained from the highest point of the first derivative plot.

### Isothermal titration calorimetry

All experiments were performed in an ITC buffer (30 mM Tris, 300 mM NaCl) while stirring at 250 rpm. The powdered stock of *N*-acetyl-d-mannosamine-6-phosphate was solubilized with the ITC buffer to a concentration of 2–5 mM before titrations. Purified *Sp*NanR was used at 200–500 μM. All titrations were performed using an initial injection of 1 μL followed by 125 identical injections of 2 μL with a duration of 4 s per injection and a spacing of 250 s between injections. The last four data points were averaged and subtracted from each titration to account for the heat of dilution (ligand into protein). Additional background experiments where buffer was titrated into the protein solution revealed no significant shift in the baseline during the measurements.

### Sedimentation velocity experiments

Sedimentation velocity experiments were performed with a Beckman Coulter XL-I with UV/visible detection optics, XL-A with a fluorescence detection system (Aviv Biomedical), or a Beckman Coulter Optima analytical ultracentrifuge with multi-wavelength UV/visible detection optics using either an An-60 Ti 4-hole or AN-50 Ti 8-hole rotor. Sample (380–460 μL) was loaded into a quartz cell with a 12-mm epon 2-channel centerpiece. Data were collected at 20°C and at 42 000 rpm, acquired in intensity mode, using radial absorbance scans at a step size of 0.001 cm (Optima) or 0.003 cm (XL-I). Diluted samples were incubated at 4°C overnight to ensure the self-associating protein molecules approached equilibrium and to minimize protein aggregation, followed by a further 2 h at room temperature as the sedimentation experiment was setup and the rotor came to temperature (20°C). SEDNTERP [46] and UltraScan [47] were used to calculate the v-bar of 0.7318 cm^3^/g for *Sp*NanR, buffer density of 1.0059 g/mL, and viscosity of 0.001034 Pa⋅s.

For the concentration series in the absence of *N*-acetylmannosamine-6-phosphate (Fig. 4 and Supplementary Fig. S4), *Sp*NanR (4.3–227.7 μM) was incubated in SEC buffer (20 mM Tris–HCl, pH 8.0, 300 mM NaCl). For the concentration series with *N*-acetylmannosamine-6-phosphate (Fig. 4 and Supplementary Fig. S5), *Sp*NanR (0.27–153.5 μM) was incubated in SEC buffer plus 2 mM *N*-acetylmannosamine-6-phosphate. Data were collected using the Optima or XL-I instruments at wavelengths outlined in Supplementary Table S3.

For the experiments shown in Fig. 5A, *Sp*NanR was serially diluted at a 2× concentration from 5 to 0.019 μM in similar fashion to above. The protein dilutions (500 μL) were mixed with 500 μL of 0.01 μM (2× final concentration) FAM-labeled *nan*E promoter sequence DNA (Supplementary Table S4) in SEC buffer. Following overnight incubation at 4°C, the samples were either prepared for the analytical ultracentrifuge experiment as above (*Sp*NanR + DNA experiment), or (*Sp*NanR + DNA + *N*-acetylmannosamine-6-phosphate experiment) each 1 mL sample was spiked with 10 μL of 200 mM *N*-acetylmannosamine-6-phosphate and incubated for four extra hours at 4°C followed by a further 2 h at room temperature as the sedimentation experiment was setup and the rotor came to temperature (20°C).

For the multiwavelength experiments in Fig. 5C and Supplementary Fig. S10, absorption spectra (210–310 nm) were collected using a Genesys S10 UV-Vis spectrophotometer (ThermoFisher Scientific) for each component: *Sp*NanR protein, *nan*E promoter DNA sequence, and SEC buffer with or without 2mM *N*-acetylmannosamine-6-phosphate. The spectra were globally fit using a nonlinear least square optimization to yield a global extinction profile for each analyte from 210–310 nm as described in Demeler B [48], which was then used to deconvolute the combined sedimentation absorbance profiles acquired in the multiwavelength centrifuge experiment, as described in Horne *et al*. [49]. All data were analyzed using UltraScan 4.0 [47]. Initially, multi-wavelength sedimentation velocity datasets from each wavelength were analyzed using two-dimensional spectrum analysis (2DSA) [50] to remove systematic noise components, and to determine boundary conditions of the sample column as reported above. Iteratively refined 2DSA models from each wavelength were used to generate a sedimentation profile for each wavelength mapped to a common time grid. Spectral deconvolution of the multiwavelength data using the molar extinction coefficient profiles of each spectral contributor generates the hydrodynamic results for each contributor. Wavelengths monitored in this experiment were 223, 225, 227, 229, 231, 233, 258, 260, 262, 276, 278, 280, and 282 nm. The data were collected and processed as described previously [49, 51].

All data were analyzed using UltraScan 4.0, release 7529 [47]. Sedimentation data were evaluated by the 2DSA [52], which affords an unbiased hydrodynamic model, where the sedimentation coefficient and frictional ratio (*f/f*_0_) can be floated independently. The models were further subjected to iterative refinement (2DSA-IT) and further subjected to genetic althorithm analyses where necessary (53). All fitting procedures were completed using the UltraScan LIMS cluster. Each 2DSA-IT model was visually assessed to ensure a good fit and a low root mean squared deviation using the FE Model Viewer utility in UltraScan (Supplementary Figs S4B and S5B). Peak integration of sample components was performed using the Genetic Algorithm Initialization Utility in UltraScan. To measure the dimer–tetramer self-association of *Sp*NanR, SEPHAT (54), was used.

The weight-averaged partial specific volume was estimated for each complex using the following equation:


\begin{eqnarray*}
{\bar {\nu}}={M^{\rm protein}}{\bar {\nu}^{\rm protein}}+M^{\rm DNA}{\bar{\nu}^{\rm DNA}}/M^{\rm protein}+M^{\rm DNA}
\end{eqnarray*}

, where M^protein^ is the molar mass of the protein component (Da), M^DNA^ is the molar mass of the DNA component (Da), ${\bar{\nu}^{\rm protein}}$ is the partial specific volume of the protein (mL/g), and ${\bar{\nu}^{\rm DNA}}$ is the partial specific volume of the DNA, which is 0.55 mL.g^−1^.

### Microscale thermophoresis

Microscale thermophoresis (MST) was carried out using a Monolith NT.115 series instrument (NanoTemper) at 20°C. FAM-labeled nucleotides identical to those used in the fluorescent AUC experiments were used as the target. Serial dilution of purified *Sp*NanR was performed in SEC buffer and PCR 8-strip tubes with individual caps (USA Scientific) following the user manual provided by NanoTemper. 2× FAM-labeled DNA oligonucleotides (40 nM) were mixed with titrated protein overnight. Samples with a final dye concentration of 20 nM were then loaded into MO-K003 Monolith NT.115 standard capillaries (NanoTemper) and were measured at 60% LED and 40% MST power. SEC buffer (20 mM Tris–HCl, pH 8.0, 300 mM NaCl) with 0.05% (v/v) Tween-20 was used for MST studies.

### Native mass spectrometry

Samples for native mass spectrometry were exchanged from the SEC buffer into 0.5 M ammonium acetate (pH 7.4) via a single-pass through a Bio-Spin *P*-6 column (Bio-Rad). Final concentrations are as quoted in Supplementary Fig. S6. Buffer-exchanged samples were then pipetted into gold-coated glass capillaries (1.0 mm outer diameter/0.75 mm inner diameter with filament), fabricated with orifice sizes of 1–5 µm, using a *P*-2000 puller (Sutter). Spectra were acquired using a Synapt XS (Waters) equipped with a 32k quad, with instrument settings optimized for the transmission of high-mass ions, and soft ionization and activation conditions as previously described [55]. The instrument was mass calibrated using CsI. Spectra were smoothed (3 × 20, mean) and mass assigned using a combination of MassLynx (Waters), standard deviation analysis [56], and UniDec [57]. Due to the salty nature of the peaks in the spectra, only those that could be unambiguously assigned have been labeled.

### Crystallography

#### Protein crystallization

Supplementary Table S7 summarizes the temperature, crystallization buffer, concentration cryoprotectant, and other parameters for the crystallization of *Sp*NanR with different ligands reported in this study. Briefly, 1 μL protein solution was mixed with 1 μL reservoir solution. The drops were incubated for 4–16 weeks before harvest. During the crystal harvest, each crystal was soaked in cryoprotectant [reservoir buffer + 20% (v/v) ethylene glycol/glycerol mix] for 2–5 s before snap-freezing in liquid nitrogen and data collection.

#### Data collection

Diffraction data were collected on the Australian Synchrotron MX2 beamline equipped with a Dectris EIGER 16M detector, and initial scaling and quality assessment performed using the XDS pipeline and AIMLESS [58, 59]. The first structure (sugar isomerase domain of *Sp*NanR + *N*-acetylmannosamine-6-phosphate, PDB ID: 8TX9) was phased experimentally (see below) and this was used as a template to solve subsequent structures by molecular replacement (PHASER [60], from the CCP4 suite of programs). The models were further refined using Refmac5 [61] and phenix.refine [62] of the CCP4 and PHENIX suites, respectively. The models were visually inspected by COOT before being deposited in the PDB [63]. Structural figures were prepared using Pymol (v 3.1.4.1).

#### Experimental phasing

Protein crystals of *Sp*NanR + *N*-acetylmannosamine-6-phosphate were added to mother liquor that had been supplemented with triiodide (10 mM) and soaked for 1–5 min; the crystals cracked if soaked longer. The crystals were washed in their mother liquor and then a cryoprotectant mix of 20% ethylene glycol/glycerol, both supplemented with triiodide, and flash frozen. We used a single wavelength anomalous diffraction (SAD) strategy. Diffraction data were collected to a resolution of 2.6–3.0 Å and processed in space group *P*2_1_. To enhance the weak anomalous signal, seven data sets were merged (see Supplementary Table S8). Initial phases were estimated using the Auto-Rickshaw SAD phasing protocol [64, 65], which incorporates SHELX [66]. The resulting partial structure, comprising only the sugar isomerase domain, was used as a starting model for the MRSAD phasing protocol in Auto-Rickshaw [64, 65], with the same anomalous data. This protocol identified 16 monomers (4 tetramers) in the asymmetric unit and located 38 triiodide molecules, corresponding to 2 triiodide sites per monomer. A single tetramer of the sugar isomerase domains was subsequently used as a template for molecular replacement with the higher resolution *I*4 dataset (*Sp*NanR + *N*-acetylmannosamine-6-phosphate, PDB ID: 8TX9), again using Auto-Rickshaw [64, 65].

#### Structure determination and refinement

Phaser [60] from the CCP4 suite of programs was used to phase structures using the sugar isomerase domain of *Sp*NanR + *N*-acetylmannosamine-6-phosphate (PDB ID: 8TX9) as the search model. The models were further refined using Refmac5 [61] and phenix.refine [62] of the CCP4 and PHENIX suites, respectively. The models were visually inspected by COOT before being deposited in the PDB [63].

For the structure with *Sp*NanR + *N*-acetylmannosamine-6-phosphate, weak density is observed for the DNA-binding domains (∼30% of the structure) for the 2.01 Å resolution structure, although the crystal has ample space for these domains. As a result, the *R*_free_ is understandably high (27.1%) despite the high-resolution data. Further crystallization trials yielded crystals that diffracted to a lower 2.95 Å resolution, but only two of the four DNA-binding domains could now be modeled (*R*_free_ = 30.1%). There was residual density for the other two DNA-binding domains, which could not be modeled.

Crystallization of the apo structure was significantly more difficult given the oligomeric heterogeneity (*K*_D_^4-2^ = 97 μM, 3.8 mg.ml^−1^), but crystals that yielded sufficient resolution data were eventually achieved (2.55 Å). Again, the *R*_free_ is rather high (31.5%), which we attribute to the structure missing one of the four DNA-binding domains and the density for the other three being weak due to flexibility (average B-factor for the DNA domains Cα atoms is 85.1 Å^2^, whereas the average B-factor for the isomerase domains is 68.3 Å^2^). We determined the structure in the space group P1 and tested various orthorhombic and monoclinic space group possibilities. In all cases, the fourth DNA-binding domain of the tetramer could not be resolved. Xtriage did not suggest twinning or pseudosymmetry.

The structure of *Sp*NanR in complex with DNA was refined using phenix.refine using NCS and Ramachandran restraints, given the low resolution (3.01 Å), and manually built using Coot. The final model has good geometry and just four Ramachandran outliers (0.4%); these residues reside in difficult to model loops. The N- and C-terminal residues were trimmed as there was little density to guide building. Density inconsistent with waters was modeled with Mg^2+^ (from the crystallization condition), which were supported by coordination number to the sugar-phosphate backbone. Additionally, four glycerol molecules (from the cryoprotection solution) and a HEPES molecule (from the crystallization condition) could be confidently placed.

## Results

### *Streptococcus pneumoniae* exhibits diauxic growth in the presence of sialic acid and glucose

Diauxic growth is a classic example of efficient microbial regulation [67, 68]. When microorganisms are provided with two carbon sources, they preferentially consume the one that supports faster growth. Only after this preferred substrate is depleted do they synthesize the enzymes needed to metabolize the second source. As a result, growth occurs in three stages: an initial exponential phase on the preferred substrate, a non-growing “diauxic lag” phase while enzymes for utilization of the second substrate are expressed, and a second exponential phase once the operon is derepressed and the secondary carbon source is utilized.

*Streptococcus pneumoniae* has been shown to exhibit diauxic growth on the substrates glucose and β-glucoside cellobiose [30]. We assessed growth in minimal media containing glucose and increasing concentrations of sialic acid (Fig. 2) and observed a biphasic growth pattern, with an initial growth phase followed by a lag phase before growth resumes, consistent with classical diauxic growth. Increasing the concentration of sialic acid enhanced the magnitude of the second growth phase, suggesting that glucose is preferentially consumed first, followed by sialic acid (Fig. 2). In this work, we focus on the molecular mechanisms that give rise to this growth pattern.

**Figure 2.**
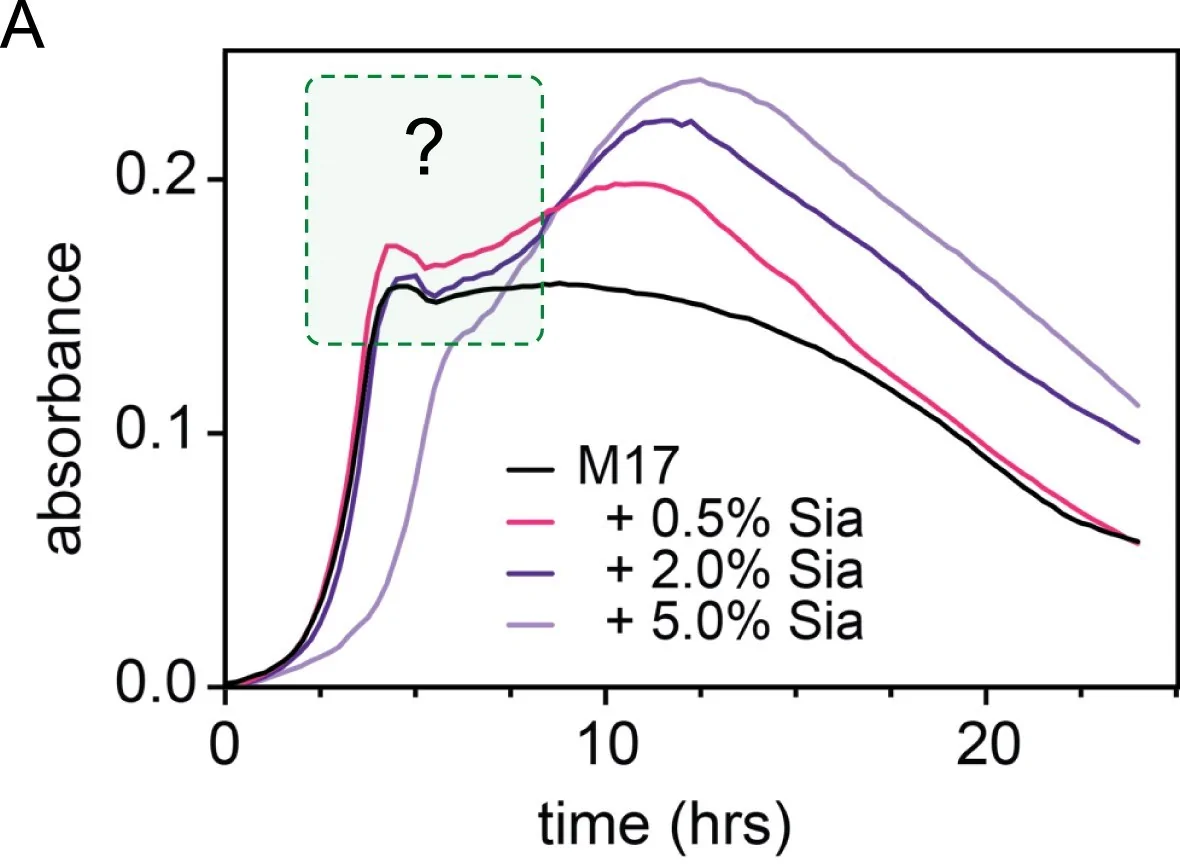
*Streptococcus pneumoniae* growth curve. (**A**) Growth of *S. pneumoniae* on M17 (no lactose) supplemented with phosphate buffered saline (PBS) buffer (control, black) or increasing quantities of sialic acid [Sia; (% w/v)]. Between 5 and 8 hours, and only in the presence of sialic acid, a lag phase is observed, followed by a secondary logarithmic growth phase. The delayed log phase observed at 5% sialic acid (light purple) may reflect a toxic effect at higher concentrations.

### The sialic acid pathway intermediate N-acetylmannosamine-6-phosphate binds *Sp*NanR

*In vivo* studies demonstrate that mannose and sialic acid promote the expression of the *S. pneumoniae nan* operon [22, 23] and that these sugars, or an intermediate metabolite within the sialic acid catabolic pathway, are the likely effector that binds to and modulates the function of *S. pneumoniae* NanR (*Sp*NanR). Recombinant, full-length *Sp*NanR (Supplementary Table S1) was expressed in *E. coli* and purified without an affinity tag, instead utilizing charge-based (ion exchange) and DNA-binding (heparin) characteristics (Supplementary Fig. S1A). To identify the *Sp*NanR effector we screened sialic acid pathway intermediates and precursors for binding using differential scanning fluorimetry (Fig. 3A, Supplementary Table S2, and Supplementary Fig. S2). Only *N*-acetylmannosamine-6-phosphate (manNAc-6-P in Fig. 3A) increases the melting temperature of *Sp*NanR (50.5°C) compared to *Sp*NanR alone (46.4°C), suggesting that binding is specific. Notably, *N*-acetylglucosamine-6-phosphate (glcNAc-6-P), which differs only by the stereochemistry of the acetyl group at the C2 position, and *N*-acetylmannosamine (manNAc), which is missing the phosphate, do not alter the melting temperature. The dissociation constant (*K*_D_) for *N*-acetylmannosamine-6-phosphate is 20.4 ± 6.0 μM (*n *= 3), measured using isothermal titration calorimetry (Fig. 3B and Supplementary Fig. S3). The binding coefficient (*N*) is 1.19 ± 0.04, suggesting that one *N*-acetylmannosamine-6-phosphate molecule binds each *Sp*NanR monomer. Together, these data support that *N*-acetylmannosamine-6-phosphate is the effector that modulates *Sp*NanR.

**Figure 3.**
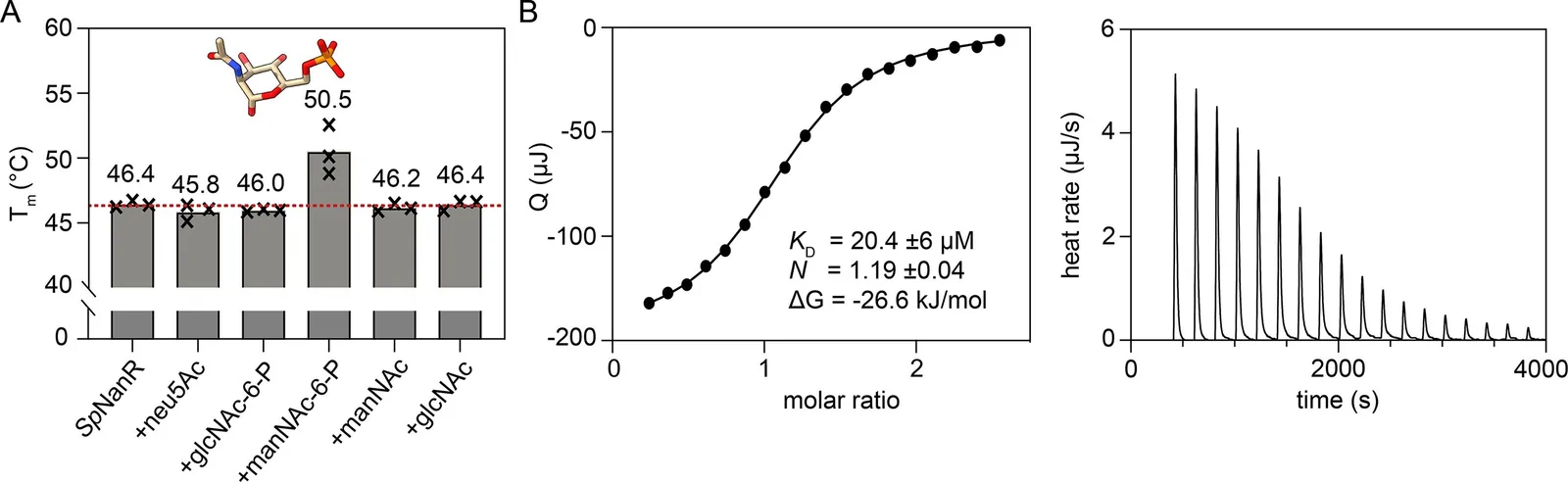
*N*-Acetylmannosamine-6-phosphate binds to *Sp*NanR. (**A**) The mean melting temperature (*T*_m_, °C) for *Sp*NanR in the presence of sialic acid derivatives. Measurements are in triplicate (black crosses). *N*-acetylmannosamine-6-phosphate (manNAc-6-P, displayed as sticks) shows a significant increase in stability, +4.1°C, relative to *Sp*NanR alone (red dash). (**B**) Representative isothermal titration calorimetry data demonstrating binding of *N*-acetylmannosamine-6-phosphate (left panel). The thermodynamic parameters are an average of three independent experiments (Supplementary Fig. S3). The heat rate (μJ/s) with corrected baseline for injections of *N*-acetylmannosamine-6-phosphate is shown (right panel).

### N*-*Acetylmannosamine-6-phosphate modulates the oligomeric state of *Sp*NanR

Recombinant *Sp*NanR elutes as a single but highly asymmetric peak by size exclusion chromatography (Supplementary Fig. S1A), indicating it may form multiple oligomeric species. To assess the oligomeric state of *Sp*NanR in solution, we undertook analytical ultracentrifugation sedimentation velocity studies across a broad range of *Sp*NanR concentrations with or without *N*-acetylmannosamine-6-phosphate. We then analyzed the data using 2DSA [52] and Monte Carlo analysis (2DSA-MC) [69], followed by van Holde–Weischet analysis [70], according to methods described in Demeler B [48].

In the absence of *N*-acetylmannosamine-6-phosphate, the van Holde–Weischet analysis (Fig. 4A, open circles) shows an increasing S value as a function of protein concentration (from ∼4.4 S to ∼5.8 S) and a shape that is diagnostic of a mass action driven self-association [71]. Differential sedimentation coefficient distributions based on 2DSA-MC across a broader concentration range (4.3–227.7 μM) shows a smaller peak ∼4.4 S at low concentrations that shifts to a larger peak at ∼5.8 S as the protein concentration increases (Supplementary Fig. S4A and Supplementary Table S3). The species at ∼4.4 S evident at the lowest concentration (4.3 μM, Supplementary Fig. S4A, dark blue trace) has a mass of 64.1 kDa determined from the 2DSA-MC distribution with a *f/f*_o_ = 1.31, which is consistent with an *Sp*NanR dimer (calculated mass from the sequence is 65.2 kDa). Native mass spectrometry corroborates the dimeric *Sp*NanR species and that the larger species evident at high protein concentrations is a tetramer (Supplementary Fig. S6A).

**Figure 4.**
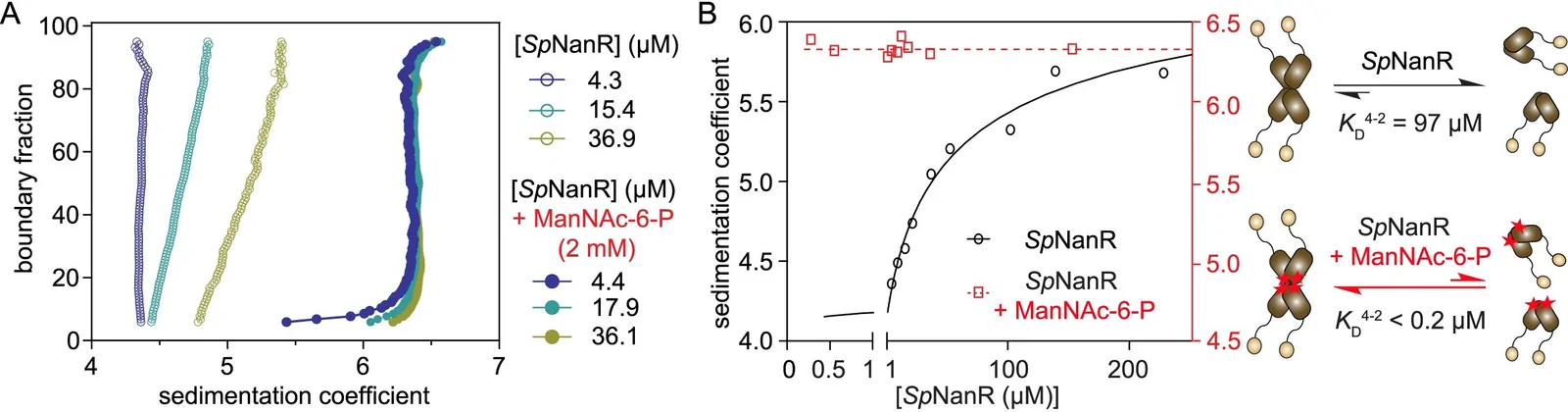
The oligomeric state of *Sp*NanR is modulated by *N*-acetylmannosamine-6-phosphate. (**A**) *Sp*NanR in the absence of *N*-acetylmannosamine-6-phosphate (open circles) self-associates between two species determined to be a dimer and tetramer. However, across the same concentration range in the presence of *N*-acetylmannosamine-6-phosphate (filled circles), only the tetramer is evident at ∼6.3 S. The van Holde–Weischet plots [70] are generated from analytical ultracentrifugation sedimentation velocity data across a range of *Sp*NanR concentrations (4.3–36.9 μM) with or without *N*-acetylmannosamine-6-phosphate (2 mM). (**B**) In the absence of *N*-acetylmannosamine-6-phosphate (black, left axis), the tetramer–dimer dissociation constant (*K*_D_^4-2^) is 97 μM (68% confidence interval = 41–234 μM). In the presence of *N*-acetylmannosamine-6-phosphate (2 mM, red, right axis), even at the lowest concentration tested (0.27 μM), *Sp*NanR is still tetrameric with a *K*_D_^4-2^ < 0.2 μM, demonstrating that *N*-acetylmannosamine-6-phosphate significantly stabilizes the tetrameric form. The weight-averaged sedimentation coefficients reported in the 2DSA-MC analysis (Supplementary Figs S4A and S5A) are plotted as a function of *Sp*NanR monomer concentration and fitted to a dimer–tetramer self-association model using SEDPHAT [54].

In the presence of *N*-acetylmannosamine-6-phosphate (2 mM), however, the van Holde–Weischet analysis shows a single species at ∼6.3 S even at the low *Sp*NanR concentration of 4.4 μM (Fig. 4A, filled circles) that is also evident in the 2DSA distribution (Supplementary Fig. S5A and Supplementary Table S3). This species at ∼6.3 S ([*Sp*NanR] = 36.1 μM, Supplementary Fig. S5A, pink trace) has a mass of 129.3 kDa (*f/f*_o_ = 1.42), which is consistent with a tetramer of *Sp*NanR (calculated mass is 130.4 kDa).

The dissociation constants (*K*_D_^4-2^) were estimated by plotting the weight-averaged sedimentation coefficients against protein concentration and fitting this to a dimer–tetramer self-association model [72] (Fig. 4B). In the absence of *N*-acetylmannosamine-6-phosphate, the *K*_D_^4-2^ is 97 μM. In contrast, in the presence of *N*-acetylmannosamine-6-phosphate (2 mM) the *K*_D_^4-2^ could not be determined as the tetrameric species dominates even at 0.27 μM, but does provide an upper boundary for the *K*_D_^4-2^ of *Sp*NanR + *N*-acetylmannosamine-6-phosphate as <0.2 μM, demonstrating that *N*-acetylmannosamine-6-phosphate binding results in at least a 2000-fold increase in the affinity for the tetramer (illustrated in Fig. 4B, right panel).

Together, these studies demonstrate that *Sp*NanR self-associates between dimeric and tetrameric species, and that the effector *N*-acetylmannosamine-6-phosphate stabilizes the tetrameric state.

### *Sp*NanR binds the nanE recognition sequence with nanomolar affinity and this is unaffected by *N*-acetylmannosamine-6-phosphate

The *S. pneumoniae* D39 genome contains three published 18 base pair near-palindromic *Sp*NanR recognition sequences that differ slightly across the *nan* cluster (Fig. 1A). In searching for the consensus sequence, 5′TCTGAAACTACTTTCAAA3′, two further putative recognition sites were identified (Fig. 1A). We determined the binding affinity of *Sp*NanR to three recognition sequences located upstream of *nan* operon-I (site 2 in Fig. 1A and B), upstream of the *nan*A gene (site 1), and the cryptic site downstream of the *sia*A operon (site 4) using MST. The recognition sequence in the promoter region of the *sia*A operon is highly similar to that of *nan* operon-I and was therefore not studied separately. Additionally, the putative site in the promoter region of *nan* operon-II was not analyzed because it is nonfunctional [22]. Despite subtle differences in their nucleotide sequence, *Sp*NanR has a similar affinity for all three (483–752 nM, Supplementary Fig. S7). We chose to focus on the 18 base pair *nanE* recognition sequence [5′TCTGAAAGTACTTTTAGA3′, doubled stranded and paired with the complementary reverse sequence indicated herein by the prime mark (T′C′…)] for subsequent studies because *nan* operon-I has been demonstrated to be activated by *Sp*NanR *in vivo* [22].

To determine the dissociation constant for *Sp*NanR and the *nan*E recognition sequence and define the effect of *N*-acetylmannosamine-6-phosphate, we titrated *Sp*NanR (0.019–5 μM) against FAM-labeled DNA (50 nM) with or without *N*-acetylmannosamine-6-phosphate and monitored the change in sedimentation coefficient using fluorescence detection analytical ultracentrifugation (Fig. 5A). The fraction of DNA bound to *Sp*NanR was determined, and in both cases the data best fitted a simple binding model with a Hill coefficient (Fig. 5B). The dissociation constant (*K*_D_) for *Sp*NanR binding to the *nan*E recognition sequence is 476 ± 75 nM, which is consistent with the MST experiment (Supplementary Fig. S7). Surprisingly, a similar dissociation constant was observed in the presence of *N*-acetylmannosamine-6-phosphate (2 mM) (549 ± 84 nM), indicating that effector binding does not significantly alter the DNA-binding affinity of *Sp*NanR for the *nan*E recognition sequence, but rather induces tetramerization.

**Figure 5.**
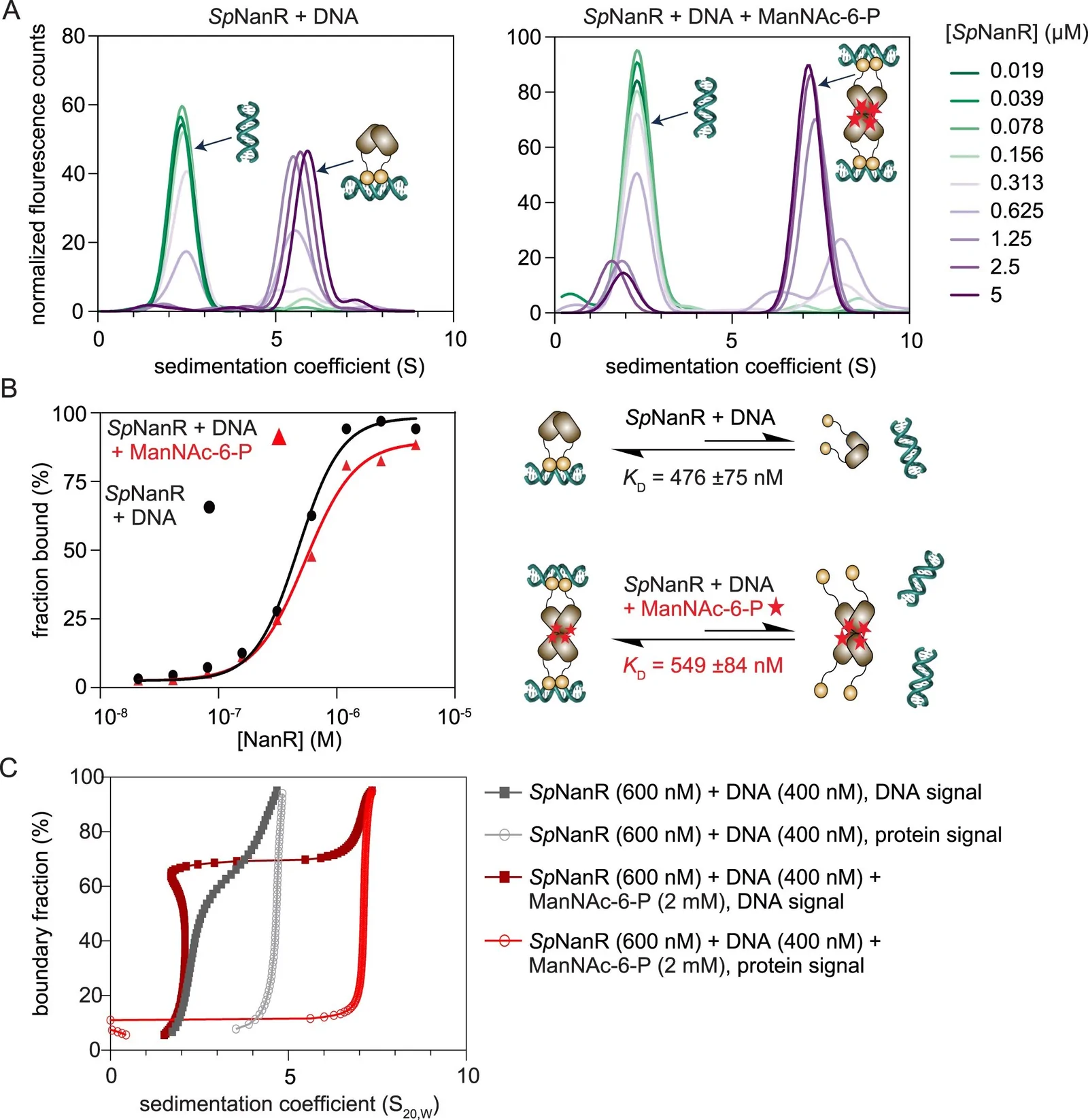
Sedimentation velocity analysis of *Sp*NanR binding to cognate DNA sequence. (**A**) Sedimentation distributions of fluorescently labeled DNA (*nan*E recognition sequence 5′TCTGAAAGTACTTTTAGA3′, 50 nM) following titration of *Sp*NanR (0.019–5 μM). The sedimentation data were evaluated by the 2DSA [52] followed by genetic algorithm analysis [53]. 2DSA distributions are shown for *Sp*NanR + DNA (left) and *Sp*NanR + DNA + *N*-acetylmannosamine-6-phosphate (2 mM, right). Fits to the data are in Supplementary Figs S8 and S9, and fit statistics are in Supplementary Table S5. (**B**) The fraction of DNA bound to protein from A (*Sp*NanR + DNA, black line; *Sp*NanR + DNA + *N*-acetylmannosamine-6-phosphate; manNAc-6-P, red line) was calculated from the data in panel (A) using UltraScan. These data were fit to a simple binding model with a Hill coefficient, plotted as a function of total *Sp*NanR concentration. The Hill coefficient (*n*) is 2.3 (95% CI = 1.75–3.11) in the absence *N*-acetylmannosamine-6-phosphate and 2.0 (95% CI = 1.50–2.68) in the presence of *N*-acetylmannosamine-6-phosphate. A schematic of the binding interactions and measured affinities for *Sp*NanR (brown) and the *nan*E recognition sequence (green), without or with *N*-acetylmannosamine-6-phosphate (red stars) is shown. (**C**) Integral distribution plots of deconvoluted multi-wavelength analytical ultracentrifugation data for *Sp*NanR (600 nM) with unlabeled *nan*E recognition sequence DNA (400 nM) in the absence (gray) or presence (red) of *N*-acetylmannosamine-6-phosphate (2 mM). The presence of *N*-acetylmannosamine-6-phosphate does not alter the amount of *Sp*NanR bound to DNA. Supplementary Fig. S10 shows a concentration series, demonstrating that the *Sp*NanR^mon^:DNA stoichiometry approaches 2:1 in the absence of *N*-acetylmannosamine-6-phosphate and 4:1 in the presence of *N*-acetylmannosamine-6-phosphate.

We corroborated these results using multi-wavelength sedimentation velocity experiments (Fig. 5C and Supplementary Fig. S10), which allowed us to monitor the DNA and *Sp*NanR sedimentation signals independently without the need for fluorescent dye. These data demonstrate that *Sp*NanR binds the *nan*E recognition sequence as both a dimer and tetramer, and that the dissociation constant for this interaction is similar with or without *N*-acetylmannosamine-6-phosphate, since the fraction bound is unchanged with the addition of the effector.

The Hill coefficient is ∼2 in both cases, suggesting there are at least two equilibria with different dissociation constants operating in this experiment. We considered two possibilities: (i) non-specific interactions between positively charged surface patches on the protein (likely within the DNA-binding domain) and the negatively charged DNA; or (ii) binding of a single DNA molecule by DNA-binding domains from two different *Sp*NanR dimers (or tetramers). Because the DNA sequence is not a perfect palindrome, the affinities of the two sites may differ, especially if binding of the first dimer/tetramer sterically hinders binding at the second site. Regardless, these data demonstrate that the effector *N*-acetylmannosamine-6-phosphate does not influence the binding affinity of *Sp*NanR for the *nan*E recognition sequence.

These experiments also allow us to estimate the stoichiometry of binding. As the *Sp*NanR concentration increases, the free DNA signal at 2.1 S decreases and a new peak at ∼5.7 S (Fig. 5A, left) or 7.2 S (right) appears, which corresponds to the *Sp*NanR–DNA complex. In the absence of *N*-acetylmannosamine-6-phosphate, the peak at ∼5.7 S corresponds to a mass of 85.3 kDa, consistent with a *Sp*NanR dimer with one DNA molecule bound (76.4 kDa). In the presence of *N*-acetylmannosamine-6-phosphate, the peak at ∼7.2 S corresponds to a mass of 144.9 kDa, consistent with a *Sp*NanR tetramer bound to either one DNA oligomer (141.8 kDa) or two DNA oligomers (153.2 kDa). These stoichiometries were corroborated by native mass spectrometry, which identifies *Sp*NanR dimer with one DNA molecule and tetramer species bound to either one or two DNA molecules (Supplementary Fig. S6B).

In summary, *Sp*NanR binds each of the three *Sp*NanR recognition sequences across the nan operons with similar affinity and requires the 18 base pair near-palindromic sequence for optimal binding. *Sp*NanR binds DNA with nanomolar affinity, though this is not altered by *N*-acetylmannosamine-6-phosphate binding.

### Structural basis of effector-binding and oligomerization

We solved crystal structures of *Sp*NanR with and without *N*-acetylmannosamine-6-phosphate to define how the effector stabilizes the tetrameric form. The structure of *Sp*NanR with *N*-acetylmannosamine-6-phosphate was initially determined using a SAD strategy (Table 1). This model was used as a template to solve subsequent structures by molecular replacement, including two further structures of *Sp*NanR with *N*-acetylmannosamine-6-phosphate (2.01 and 2.95 Å), apo-*Sp*NanR (2.55 Å), and *Sp*NanR complexed with DNA (next section).

**Table 1.** Data collection and refinement statistics for *Sp*NanR crystal structure

	*Sp*NanR + manNAc-6-P + triiodide*	*Sp*NanR + manNAc-6-P	*Sp*NanR + manNAc-6-P	*Sp*NanR	*Sp*NanR + DNA
**Data collection**					
Wavelength (Å)	1.458 644	0.953 649	0.953 732	0.953 739	0.953 739
Space group	*P*2_1_	*I*4	*C*222	*P*2_1_2_1_2_1_	*P*22_1_2_1_
Unit cell parameters [a, b, c (Å)]	147.3, 84.4, 148.0 (β = 90.2°)	147.6, 147.6, 82.3	116.9, 223.7, 52.7	53.2, 109.2, 202.4	57.9, 159.4, 171.8
Resolution range (Å) (outer shell)^**^b^**^	49.3–2.65 (2.71–2.65)	42.2–2.01 (2.08–2.01)	47.7–2.95 (3.06–2.95)	45.9–2.55 (2.64–2.55)	48.0–3.01 (3.12–3.01)
No. of unique reflections	207 452 (14 687)	58 719 (5722)	133 994 (13 126)	39 440 (3859)	30 086 (3003)
Multiplicity	21.0 (3.5)	13.5 (13.8)	8.9 (8.9)	10.1 (10.1)	9.5 (10.0)
Completeness (%)	99.6 (95.3)	99.8 (95.7)	99.9 (99.9)	97.6 (98.0)	92.8 (100.0)
*I/σ(I)*	13.0 (1.0)	16.8 (1.6)	7.4 (0.9)	9.16 (0.77)	10.0 (1.2)
CC_(1/2)_	0.999 (0.498)	0.999 (0.777)	0.994 (0.355)	0.998 (0.61)	0.99 (0.491)
CC_anom_	31(0)	-	-	-	-
*R*_merge_	0.17 (1.04)	0.080 (1.187)	0.2461 (2.364)	0.188 (2.469)	0.170 (2.249)
**Refinement statistics**					
No. of reflections used	-	58 704	15 052	38 521	30 086
No. of protein atoms	-	5 991	3 712	7 863	8 685
No. of water molecules	-	184	0	21	1
No. of ligand atoms	-	100	51	64	14
No. of DNA atoms	-	0	0	0	1 476
*R*_work_ (%)	-	21.0	25.0	26.2	19.4
*R*_free_ (%)	-	27.1	30.1	31.2	22.2
Reflections used for *R*_free_	-	2 832 (306)	1 767 (100)	1 898 (194)	1 460 (169)
Ramachandran (favored, allowed, disallowed)	-	98.2, 1.8, 0.0	96.29, 3.71, 0.0	96.6, 3.3, 0.1	96.2, 3.5, 0.3
RMS bond lengths (Å)	-	0.015	0.006	0.006	0.011
RMS angles (°)	-	1.490	0.87	0.83	1.21
Overall B-factor (protein, waters)	-	60.85	72.91	74.3	85.16
Clashscore (PHENIX)	-	9.34	13.0	18.9	10.7
PDB id	-	8TX9	8V4V	8V5F	9DQN

a This is a merged dataset described in Supplementary Table S8.

b Parameters in brackets indicated the highest resolution shell.

The *Sp*NanR monomer comprises two domains connected by a linker region of 11 residues (Fig. 6A). The N-terminal DNA-binding domain comprises helices α1–α5 and adopts the well-characterized helix–turn–helix motif, which is common throughout prokaryotic and eukaryotic DNA binding proteins [73]. The C-terminal sugar isomerase domain comprises an α/β-fold, with five parallel β-strands forming the core flanked by five of eight α-helices creating an αβα sandwich structure. Alignment of ligand-free monomers, however, highlights the alternative conformational arrangements of the DNA-binding domains relative to the sugar isomerase domains, likely due to flexibility in the linker region (Supplementary Fig. S11A). This linker flexibility is further evidenced by the observation that only the backbone of the linker could be confidently modeled in the apo structure (Supplementary Fig. S11B). The five DNA-binding domains across the two structures align well (r.m.s.d. = 0.44–0.75 Å, Supplementary Fig. S11C) and the C-terminal domains also show strong structural agreement across all structures (r.m.s.d. = 0.20–0.35 Å, Supplementary Fig. S11D). Further, the sugar isomerase domain, including the placement of the ligand binding pocket, is conserved across the four RpiR transcriptional regulator structures reported (Supplementary Fig. S11E). We note that this domain aligns well with the sugar isomerase domain of glucosamine-6-phosphate-synthetase (GlmS, PDB ID: 1MOR, r.m.s.d. = 2.6 Å, Supplementary Fig. S11F), which is known to isomerize fructose-6-phosphate to glucosamine-6-phosphate [74] and is likely an evolutionarily related domain.

**Figure 6.**
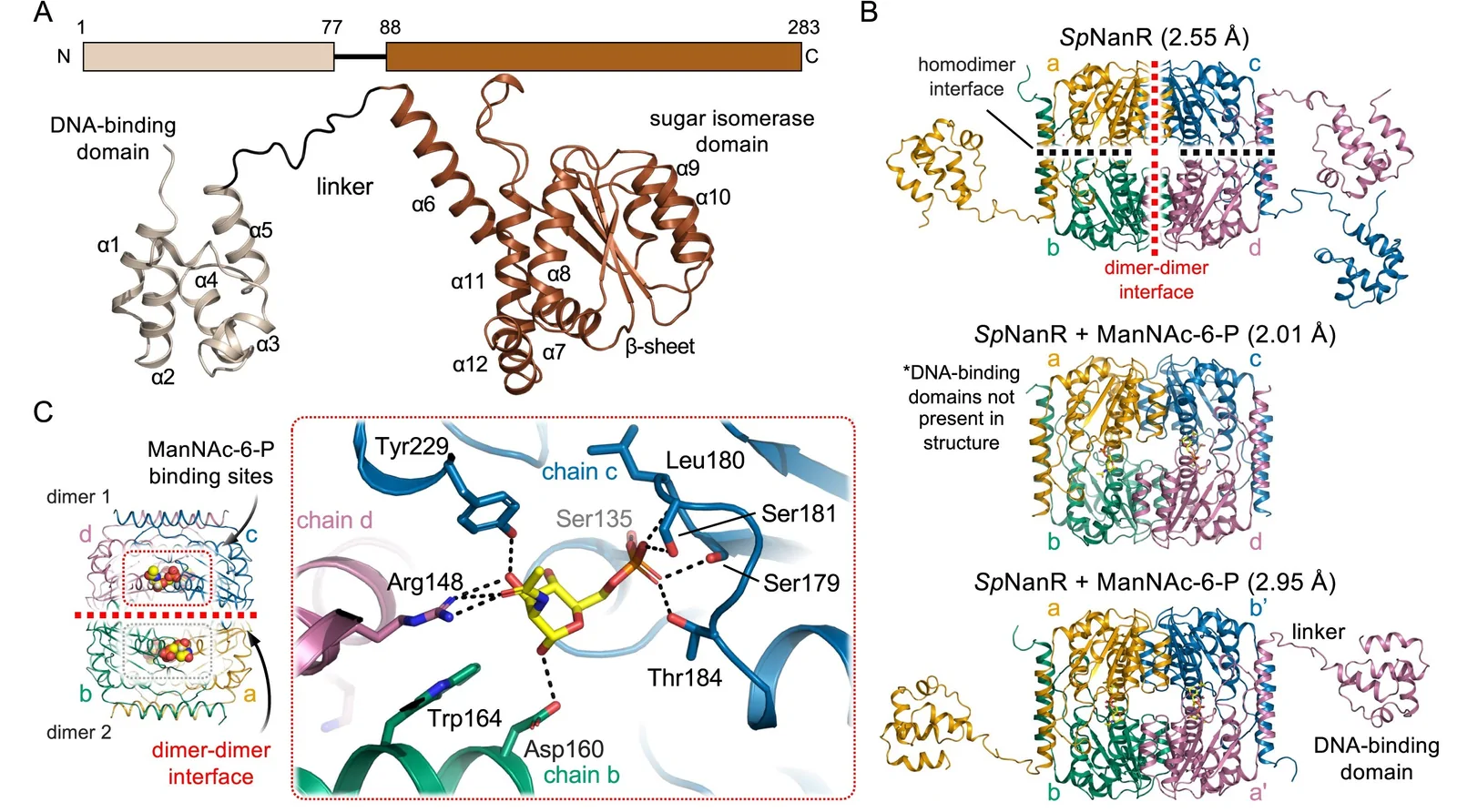
Structure of *Sp*NanR and the *N*-acetylmannosamine-6-phosphate binding site. (**A**) The *Sp*NanR monomer is made up of two domains; an N-terminal DNA-binding domain (light brown) and a C-terminal sugar isomerase domain (dark brown) fused by a flexible 11 amino acid linker region (black). (**B**) Crystal structures of *Sp*NanR assemble into a tetramer with and without *N*-acetylmannosamine-6-phosphate. Each monomer is colored and annotated a–d. Two interfaces are formed: a dimer interface (black dotted lines) and a dimer–dimer interface (red dotted lines), calculated via PDBePISA. (**C**) *N*-Acetylmannosamine-6-phosphate binds close to and bridges across the dimer–dimer interface (dotted lines).

The *Sp*NanR tetramer adopts a dimer-of-dimers architecture with D2 point-group symmetry [75]. Tetramerization is mediated by two extensive interfaces involving the sugar isomerase domains; a primary dimer interface between monomers a/b and c/d (Fig. 6B, black dashed line, buried surface area = ∼855 Å^2^), and a secondary dimer–dimer interface between monomers a/c and b/d, which forms the tetramer (red dashed line, buried surface area = ∼835 Å^2^). We note that neither the DNA-binding domains nor the linker regions contribute to oligomerization. Instead, the flexible linker regions (residues 77–88) position the DNA-binding domains distal to the central sugar isomerase domains that form the tetramer (Fig. 6B and Supplementary Fig. S11B).

We then sought to examine whether the tetrameric configuration observed in our crystal structure resembled the tetramer in solution using small angle X-ray scattering experiments (SAXS; statistics in Supplementary Table S9). In-line size exclusion chromatography demonstrated that *Sp*NanR is polydisperse, with both tetrameric and dimeric populations (Supplementary Fig. S13A). In contrast, *Sp*NanR in the presence of *N*-acetylmannosamine-6-phosphate (2 mM) is monodisperse and exclusively tetrameric (Supplementary Fig. S13B). The maximum particle dimension (*D*_max_) of *Sp*NanR with or without *N*-acetylmannosamine-6-phosphate was ∼165 Å from the *P*(*r*) analysis (Supplementary Fig. S13C), which is consistent with the *D*_max_ measured from the crystal structures (∼160 Å). The Guinier analysis produced a linear plot for both datasets supporting that each sample is free from aggregation or inter-particle interference (Supplementary Fig. S13D). Next, we evaluated whether the extended conformation observed in the crystal structures, or an alternative compact conformation in which the DNA-binding domains interact directly with isomerase domains (seen in homologues), could be detected in solution. Using CRYSOL, the theoretical scattering profile of the extended conformation was best fit to the experimental SAXS data with *N*-acetylmannosamine-6-phosphate (purple, *χ*^2^ value of 0.38; Supplementary Fig. S13E), supporting that our structure matches the in-solution structure and is not an artifact of the crystal lattice. We note the tight conformation of *N*-acetylmannosamine-6-phosphate (green, *χ*^2^ value of 0.48) has significant deviation at low *q*, contributing to a poorer overall fit. Further, the Kratky analysis suggested that *Sp*NanR has two domains connected via a flexible linker, with or without *N-*acetylmannosamine-6-phosphate (Supplementary Fig. S13F), which we observe in our electron density maps (Supplementary Fig. S11B) and the bimodal charge state distributions by native mass spectrometry (Supplementary Fig. S6A) [76, 77].

Four *N*-acetylmannosamine-6-phosphate molecules bind in identical pockets near the dimer–dimer interface (Fig. 6C and Supplementary Table S6). The tetrameric sugar isomerase domains from all three structures overlay closely (Cα r.m.s.d. = 0.30–0.46 Å) and the dimer–dimer interface remains largely unchanged between the unbound and *N*-acetylmannosamine-6-phosphate bound structures (Supplementary Fig. S13E and F), indicating that *N*-acetylmannosamine-6-phosphate does not stabilize the tetramer by rearranging this interface. Instead, each *N*-acetylmannosamine-6-phosphate molecule bridges three monomers across the tetrameric interface, thereby stabilizing the tetrameric state (Fig. 6C). The phosphate group coordinates Ser135, Ser179, the Leu180 backbone, Ser181, and Thr184 from one monomer, while the sugar moiety interacts with Arg148 and Tyr229 from the second monomer within the dimer and with Asp160 from the third monomer across the dimer–dimer interface (Fig. 6C inset, Supplementary Fig. S13A, and Supplementary Table S6). Arg148 coordinates the C2 *N*-acetyl and C3 hydroxyl groups, explaining why *Sp*NanR does not bind *N*-acetyl glucosamine-6-phosphate (Fig. 2A), whose *N*-acetyl group adopts a different stereochemical orientation. Notably, Arg148 also participates in a cation–π–anion stacking interaction with Trp164 and Asp160 across the dimer–dimer interface.

To examine the contribution of binding site residues to effector binding and oligomerization, we prepared recombinant *Sp*NanR variants containing site-specific alanine substitutions and evaluated each variant’s thermal stability by differential scanning fluorimetry. Variants included a triple mutant *Sp*NanR^S179A/S181A/T184A^, which probes phosphate coordination, as well as *Sp*NanR^R148A^ and *Sp*NanR^D160A^, which probe binding of the *N*-acetyl and sugar hydroxyl groups and the role of the cation–π–anion stacking interaction across the dimer–dimer interface, respectively (Fig. 6C). All *Sp*NanR variants exhibited melting temperatures that were comparable with wild type (ranging from 49°C to 51°C; Supplementary Fig. S15B), suggesting they are correctly folded. Upon incubation with *N*-acetylmannosamine-6-phosphate, wild-type *Sp*NanR showed an increase in thermal stability (from 50.8°C to 63.0°C), consistent with effector binding. *Sp*NanR^D160A^, which bridges the dimer–dimer interface, exhibited a comparable increase, indicating that this residue is less important for effector binding. Conversely, *Sp*NanR^S179A/S181A/T184A^ and *Sp*NanR^R148A^ showed only marginal changes in thermal stability in the presence of *N*-acetylmannosamine-6-phosphate, suggesting that effector binding is abolished.

We next tested whether these *Sp*NanR variants affected oligomeric state using sedimentation velocity experiments. As expected, *N*-acetylmannosamine-6-phosphate does not stabilize the tetrameric state of *Sp*NanR^S179A/S181A/T184A^, consistent with thermal stability data indicating that the effector does not bind this variant (Supplementary Fig. S15C). Interestingly, the *Sp*NanR^D160A^ variant is exclusively a dimer in the absence of *N*-acetylmannosamine-6-phosphate, whereas wild-type *Sp*NanR shows evidence of both dimeric and tetrameric forms at the same concentration (15 μM, Supplementary Fig. S15D). This supports the notion that *Sp*NanR^D160A^ destabilizes the tetrameric form. However, in the presence of *N*-acetylmannosamine-6-phosphate, the *Sp*NanR^D160A^ variant behaves similarly to wild type, consistent with the finding that it still binds *N*-acetylmannosamine-6-phosphate and that this sufficiently stabilizes the tetrameric form. In contrast, *Sp*NanR^R148A^ is exclusively dimeric at the same concentration as wild type even in the presence of *N*-acetylmannosamine-6-phosphate (Supplementary Fig. S15E). This suggests that the cation–π–anion stacking interaction across the dimer–dimer interface is critical for tetramer formation and subsequent stabilization by *N*-acetylmannosamine-6-phosphate.

Because *Sp*NanR^R148A^ is exclusively dimeric, this provided an opportunity to determine how the dimeric unit is assembled (the tetrameric form leads to three possibilities depending on the interface that dimerizes). To do this, we collected SAXS data for *Sp*NanR^R148A^ and compared these data to the back calculated scattering profiles of models for each of the possible dimer assemblies using CRYSOL (Supplementary Fig. S14). This analysis demonstrates that the hand-in-hand dimer best fits the data (purple, *χ*^2^ = 0.65), further supporting that neither the DNA-binding domains nor the linker regions contribute to oligomerization.

Collectively, crystal structures of *Sp*NanR with and without *N*-acetylmannosamine-6-phosphate reveal how *Sp*NanR oligomerizes, show how the effector bridges the dimer–dimer interface to stabilize the tetramer, and explain why effector binding does not affect DNA binding.

### *Sp*NanR recognizes the palindrome through an “indirect readout” mechanism

We determined the structure of *Sp*NanR in complex with the *nan*E recognition sequence (5′TCTGAAAGTACTTTTAGA3′, underline representing a break in the palindrome) to define how *Sp*NanR engages a specific DNA sequence. Crystals of the complex diffract to 3.01 Å and the final structure has good global statistics (*R*_free_ = 22.2%) and geometry (Table 1). All amino acids (except three residues at the N- and C-terminal) and all nucleotides could be built into the electron density. This structure is without *N-*acetylmannosamine-6-phosphate, since its addition failed to yield crystals that diffracted despite significant effort. The asymmetric unit contains two *Sp*NanR dimers, and two *nan*E recognition sequence DNA molecules (Fig. 7A). The two dimers are loosely connected but offset in what appears to be crystal contacts at the same dimer–dimer interface used to form the tetramer.

**Figure 7.**
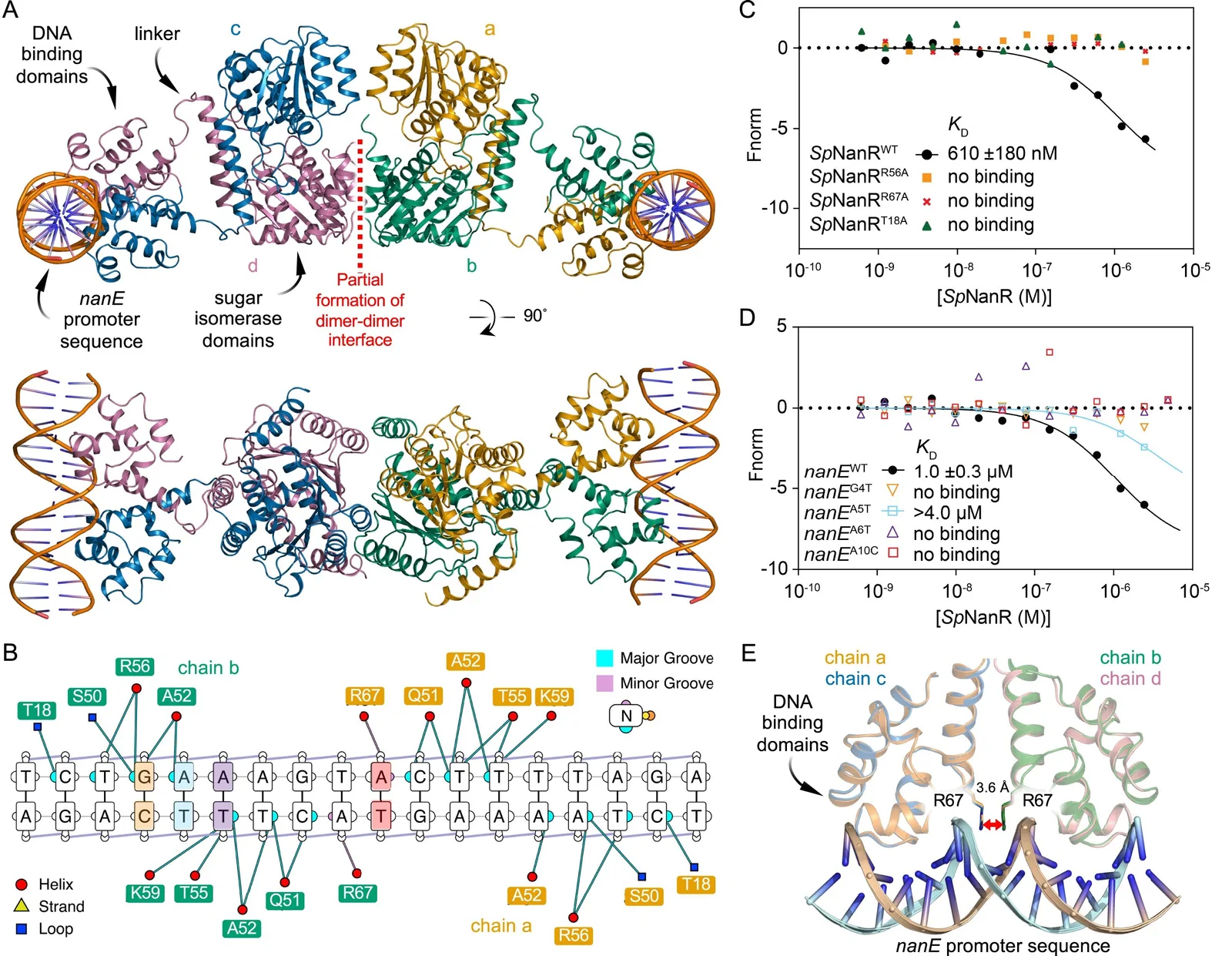
Structure of *Sp*NanR in complex with the *nan*E recognition sequence. (**A**) The protein–DNA complex crystallized with two *Sp*NanR dimers in the asymmetric unit, each bound to an 18 base pair dsDNA *nan*E recognition sequence (5′TCTGAAAGTACTTTTAGA3’). Each monomer is individually colored. (**B**) *nan*E recognition sequence and *Sp*NanR amino acid interactions are depicted. Nucleotides that are mutated are colored (G4, A5, A6, and A10). Their binding affinity is tested in panel (D). (**C**) MST results when titrating *Sp*NanR and variants (10–5 000 nM) against the native FAM-labeled *nan*E recognition sequence and mutated variants (20 nM). (**D**) MST results when titrating *Sp*NanR (10–5000 nM) against native and mutated FAM-labeled *nan*E recognition sequences (20 nM). (**E**) View of the *Sp*NanR DNA-binding domains from the *Sp*NanR dimer binding to the 18 base pair *nan*E recognition sequence (sense strand = cyan, anti-sense = tan), highlighting the unique binding contributions from each R67 (denoted as sticks) residue in the two *Sp*NanR monomers.

In each dimer, the DNA-binding domains engage one DNA molecule and are spatially separated from the sugar isomerase domains by the disordered linker regions. Aligning the two dimers in the asymmetric unit shows that the dimeric sugar isomerase domains align well (r.m.s.d. = 0.47 Å over 361 Cα), however the DNA-binding domains and *nan*E recognition sequence DNA components are then offset (Supplementary Fig. S16A). This suggests that although the DNA-binding domains interact with the *nan*E recognition sequence in the same manner, they are conformationally flexible relative to the core sugar isomerase domains. An overlay of the DNA-binding domains demonstrates that each dimer engages the DNA sequence in the same way (Supplementary Fig. S16B). Though the *nan*E recognition sequence maintains the overall hallmarks of B-DNA conformation (right-handed double helix with base pairs perpendicular to the helix axis), it is distorted from a straight strand when bound to *Sp*NanR (Supplementary Movie S1).

Multiple experiments suggest that, in solution, *Sp*NanR bound to DNA and *N-*acetylmannosamine-6-phosphate adopts an extended and flexible conformation. First, we collected SAXS data with in-line size exclusion chromatography for the *Sp*NanR:*nan*E recognition sequence:*N-*acetylmannosamine-6-phosphate complex (Supplementary Fig. S17A and Supplementary Table S9). The *D*_max_ from these data is ∼178 Å, which is consistent with the longest measured distance from the crystal structure (∼175 Å) and the Kratky plot has two distinct peaks consistent with multiple, independent domains (Supplementary Fig. S17B and C). Using CRYSOL, we generated theoretical scattering profiles based on the crystal structure and additional models that include one or two DNA molecules. When compared against experimental SAXS data, the model of the tetramer with two DNA molecules bound and extended away from the isomerase domains best fit the data (blue, *χ*^2^ value of 0.085; Supplementary Fig. S17D). Next, native mass spectrometry data report the presence of extended/flexible species when in the presence of DNA and *N-*acetylmannosamine-6-phosphate (Supplementary Fig. S6B). Finally, attempts to determine the structure of the *Sp*NanR:*nan*E recognition sequence:*N*-acetylmannosamine-6-phosphate complex using single particle cryo-electron microscopy were unsuccessful due to particle heterogeneity (data not shown). Notably, while the central sugar isomerase domains could be resolved, the DNA-binding domains in complex with DNA could not be resolved. Taken together, these data support a flexible domain architecture in which the DNA-binding domains are still decoupled from the isomerase domains, consistent with the crystal structure, although DNA binding likely constrains the DNA-binding domains.

*Sp*NanR engages the *nan*E recognition sequence in a manner that explains the sequence specificity. Each monomer within the *Sp*NanR dimer interacts symmetrically with the major DNA groove via a conserved helix–turn–helix motif. A survey of the interactions between *Sp*NanR and the *nan*E recognition sequence is shown in Fig. 7B and is consistent across both dimers in the asymmetric unit. As the *nan*E recognition sequence is an imperfect palindrome, contacts made by each DNA-binding domain are similar but not identical. The recognition helix of *Sp*NanR (residues 50–61) nestles into the major DNA groove above the A–T track. Recognition and binding to specific nucleotide sequences often involves the formation of hydrogen bonds between amino acid side chains and specific bases in the major groove—a process known as the “direct readout” mechanism [78]. The recognition helix forms minor, long distance hydrogen bonds with bases in the major groove, instead largely interacting with the sugar phosphate backbone (Fig. 6B and Supplementary Fig. S16C). Arg56 weakly interacts with the N7 nitrogen of the base at position 4 (guanine) or position 15′ on the reverse strand (adenine) at ∼3.2 Å (average over four monomers in the asymmetric unit); these positions break the palindrome for the *nan*E recognition sequence. It also interacts with the phosphate backbone (distance ∼3.0 Å). Substituting Arg56 with alanine (*Sp*NanR^R56A^) results in no detectable binding to the *nan*E recognition sequence compared to *Sp*NanR^WT^ (Fig. 7C, orange box).

Modeling suggests that mutating guanine at position 4 or adenine at position 15 to the pyrimidines thymine or cytosine introduces a steric clash, partly explaining the sequence specificity at this position. Indeed, mutagenesis of the guanine cytosine base pair at position 4/15′ to thymine adenine (*nan*E^G4T^) abolishes binding, as demonstrated by MST (Fig. 7D, orange open triangle). Together, these results suggest that although the interaction between Arg56 and the base is weak, it is nonetheless essential for binding the *nan*E recognition sequence. Thr55 interacts exclusively with the backbone phosphate at position 12 of the sequence (∼2.7 Å), while Lys59 and Ser50 contact the backbone phosphate at positions 13 (∼2.8 Å) and 4 (∼2.6 Å), respectively. Ala52 projects into the major groove, but its methyl side chain does not make any direct connections with the bases. Although water-mediated interactions are possible, none were observed in the major groove at 3.01 Å resolution, even though waters interacting with DNA are visible elsewhere. Outside of the recognition helix, the Thr18 side chain and the Leu20 main chain amide contact the phosphate backbone in the major DNA groove. Substitution of Thr18 with alanine (*Sp*NanR^T18A^) abolishes binding (Fig. 7C, green triangle). In addition, the main chain amides of Tyr66 and Arg67 form hydrogen bonds with the phosphate of the nucleotide at position 12 (∼2.6 Å and ∼3.5 Å, respectively).

Because the *nanE* recognition sequence is near-palindromic, we tested for possible statistical disorder by re-refining the structure with the opposite DNA register (5′TCTAAAAGTACTTTCAGA3′). When using the exact refinement parameters, the statistics were marginally worse [*R*/*R*_free_ = 0.1939/0.2217 (original) versus 0.1944/0.2223 (opposite) at 3.01 Å] and no obvious *F_o_–F_c_* density difference is observed. These data do not strongly discriminate between registers and therefore statistical disorder cannot be excluded. We therefore interpret the Arg56–base contact cautiously but note that Arg56 and G4 are functionally required for binding (as described above) and there is density for the side chain-nucleotide interaction as illustrated in the composite omit map included in Supplementary Fig. S19. Future studies employing brominated DNA derivatives or explicit multi-state occupancy refinement may help resolve whether the near-palindromic recognition sequence exhibits partial register disorder within the crystal lattice.

A distinctive feature is the insertion of Arg67 side chains from each *Sp*NanR monomer into the minor DNA groove (Fig. 7E). The guanidinium groups π-stack ∼3.6 Å apart (averaged across the two dimers) and each forms a weak hydrogen bond with the N3 nitrogen of adenine at position 10 (∼3.5 Å). Both side chains also contact the backbone phosphates at positions 10 (∼3.8 Å) and 11 (∼3.4 Å), respectively. Substitution of Arg67 with alanine abolishes binding (*Sp*NanR^R67A^, Fig. 7C, red cross), indicating that this interaction is essential for engagement of the *nan*E recognition sequence. Arginine residues are commonly observed interacting with the minor groove [79]. However, a survey of helix–turn–helix DNA-binding domains in which an arginine engages the minor groove [79] suggest that the π-stacked arginine conformation observed here are unique, expanding the repertoire of interactions that recognize the minor groove of B-DNA.

Aligning the *nan*E recognition sequence with an idealized B-DNA molecule with the same sequence shows it is distorted (r.m.s.d. = 1.4 Å aligning all 18 base pairs, Supplementary Movie S1), suggesting that recognition and specificity may be due to an “indirect readout” mechanism [80]. This is especially relevant because each monomer of *Sp*NanR makes only two long-range interactions with the base pairs: Arg56 (∼3.2 Å) and Arg67 (∼3.5 Å).

The minor groove width is significantly deformed upon *Sp*NanR binding (Fig. 8A)—the width is compressed where engaging the recognition helix (8.6 Å, measured from the phosphate +4 bases of the opposing strand) and widened at nucleotides 9 and 10 where the two Arg67 insert (10.7 Å, Fig. 8B). The minor groove width for an idealized B-DNA molecule is 11.7 Å. Base-pair stacking is most perturbed at this position, where the TA dinucleotide (positions 9 and 10) is significantly misaligned (helical twist ∼50° versus ∼36° for ideal B-DNA; Fig. 8C) and instead appears more favorably stacked with the adjacent C and G bases (helical twist ∼26°). Arginine residues are enriched within the minor groove and commonly interact with adenine and thymine, especially where the minor groove narrows [79]. Although substitution of adenine at position 10 to cytosine in the *nan*E recognition sequence would be tolerated—since it does not introduce a steric clash and could support hydrogen bonding with Arg67—it nonetheless abolishes binding (*nan*E^A10C^, Fig. 7D, open red box). TpA steps, as found in the *nan*E recognition sequence at positions 9/10 and 15/16, increase flexibility in B-DNA molecules [81–83], reviewed in Marin-Gonzalez *et al*. [84]. We reason that the mutation *nan*E^A10C^ breaks the TpA step and stabilizes the DNA architecture such that *Sp*NanR cannot recognize or engage a distorted structure.

**Figure 8.**
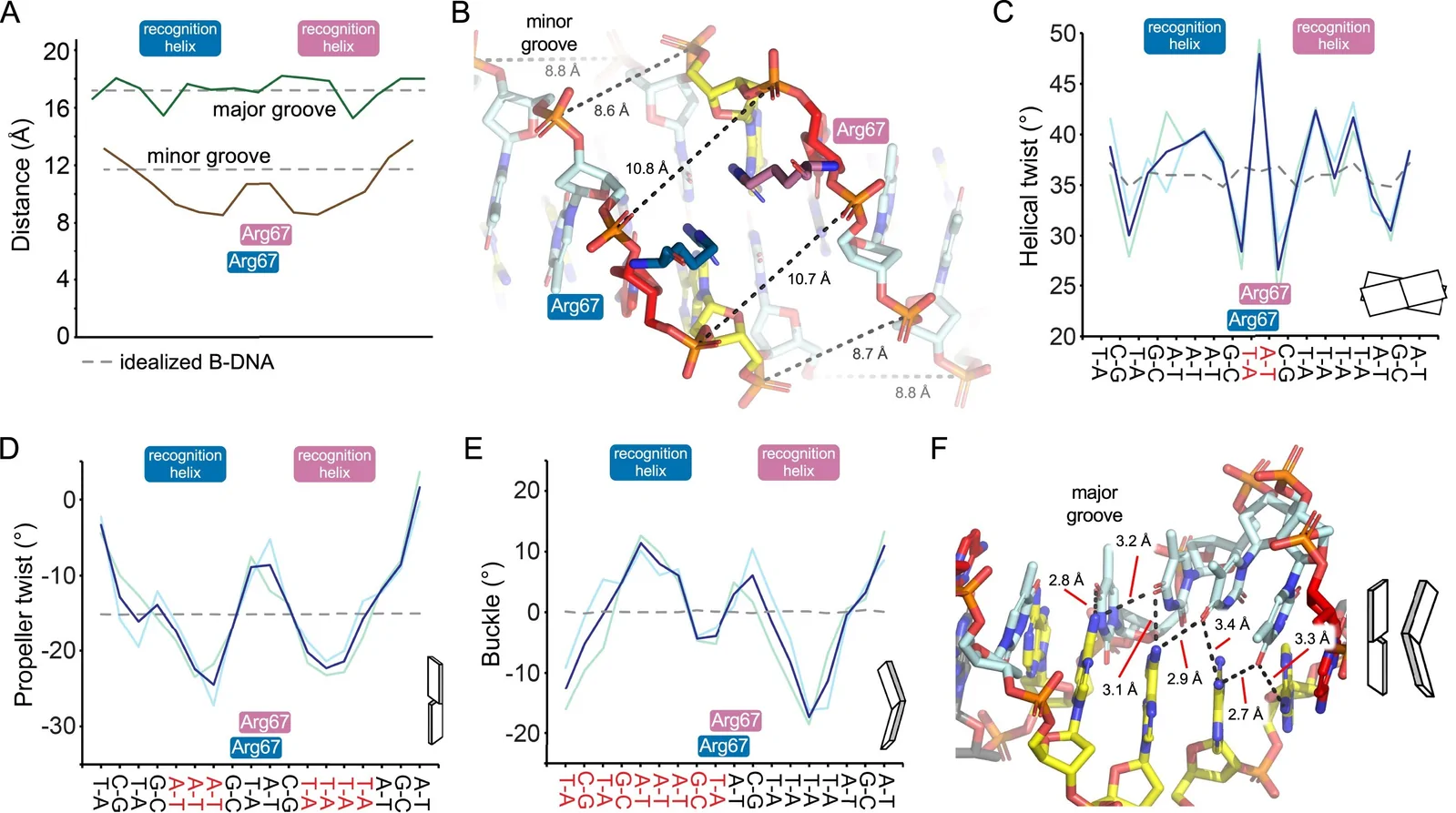
The *nan*E recognition sequence adopts a distorted B-DNA architecture when bound by *Sp*NanR. (**A**) Major and minor groove widths across the *nan*E recognition sequence bound to *Sp*NanR. (**B**) The distances across the minor groove, measured from opposing backbone phosphate, at the point of Arg67 insertion. (**C**) Degree of helical twisting between base pairs. (**D**) Degree of propeller twist within base pairs. (**E**) Degree of buckle within base pairs. (**F**) Disruption of the base pairing within the major groove below the recognition helix. For panels (A), (C), (D), and (E), the dotted line represents the norm for an idealized B-DNA molecule of the same sequence and length. A cartoon depiction of each measurement is shown at the bottom right of each plot. The approximate positions of the recognition helices and Arg67 from each monomer are also shown. Based on the calculated coordinate error in the *Sp*NanR–DNA crystal structure the following error can be estimated; major and minor groove widths of ±0.7 Å, helical twists of ±3°, and propeller twist and buckle of ±3°–5°.

Although the major groove width is close to ideal (Fig. 8A), the bases are buckled and twisted, affecting the pattern of base pairing. The alignment of the base pairs (propeller twist) is especially altered where the recognition helices bind and where the Arg67 side chains insert (Fig. 8D). In addition, the base pairs are significantly buckled through the A–T track below the recognition helices (Fig. 8E). This causes a deviation away from the Watson–Crick pattern in an idealized B-DNA molecule, such that the bases also form hydrogen bonds with opposing, but neighboring, bases (Fig. 8F).

In summary, the DNA-binding domains of *Sp*NanR remain disengaged from the sugar isomerase domains when bound to the *nan*E recognition DNA sequence, explaining why *N*-acetylmannosamine-6-phosphate does not affect DNA binding. The *Sp*NanR recognition helix binds the major groove, as expected, but with only one long-range interaction with a base each side of the near palindromic sequence. Two arginine residues (Arg67 from each domain) insert into the minor groove, form an unusual π-stack conformation, each make an additional long-range hydrogen bond with a base, and together disrupt the minor groove width. These observations suggest that specificity is achieved through recognition of a sequence-dependent distorted B-DNA structure—the “indirect read-out” mechanism—while affinity is primarily driven by interactions between the DNA-binding domain and the phosphate backbone.

## Discussion

The import and catabolism of sialic acid is an adaptive mechanism for many bacterial pathogens (recent reviews [4, 14, 85, 86]). For *S. pneumoniae*, the link between sialic acid utilization and both colonization and pathogenesis is well-established. For example, addition of exogenous sialic acid leads to a significant increase in *nan* cluster transcription in naturally colonizing *Streptococci* [13], and it has been long known that sialidase enzymes (in this context the *sia*A and *nan*B genes), which cleave sialic acids from host glycoconjugates, are virulence factors for *S. pneumoniae* pathogenesis [14]. Disrupting genes that import and metabolize sialic acid results in loss of fitness during colonization for many pathogenic bacteria [14, 85, 86], including *S. pneumoniae* [87]. When in the presence of *N*-acetylmannosamine or sialic acid, *S. pneumoniae* upregulates expression of the machinery needed to catabolize sialic acid encoded by the *nan* gene cluster [23], and knocking out the *nan*R gene abolishes this response [22]. The *nan* gene cluster comprises three operons (*nan* operon-I, *nan* operon-II, and *sia*A) that generate three transcripts. Three *Sp*NanR recognition sites were previously reported across the cluster [22] and we identify two additional sites. Among the five identified sites, three are located within the promoter regions of their respective operons, while the remaining two are positioned at cryptic loci with poorly defined regulatory functions (Fig. 1A). Given the importance of sialic acid uptake for bacterial colonization and pathogenesis, including for *S. pneumoniae*, we set out to define the molecular mechanism by which *S. pneumoniae* senses sialic acid in the environment and controls the expression of the genes required for its metabolism. Here, we propose a mechanism by which *Sp*NanR regulates sialic acid uptake that is consistent with our structural and biophysical data, and the previously reported functional data [22].

### Architecture and regulation across the *nan* gene cluster

The *nan* operon-I and *sia*A operon transcripts are upregulated when *S. pneumoniae* is grown on sialic acid-rich media [22]. Their promoter regions are similar in architecture, with functional *Sp*NanR recognition sequences ∼55 base pairs upstream of the −35 box (Supplementary Fig. S18A–C). This general structure is conserved across *S. pneumoniae* strains and other *Streptococcus* bacteria, especially for the *nan* operon-I (Supplementary Table S10). We demonstrate that *Sp*NanR binds the *nan* operon-I promoter region upstream of the RNA polymerase binding site, consistent with its role as an activator of transcription. Although we did not study the *Sp*NanR binding site within the *sia*A operon promoter site, it is very likely that it functions identically to the *nan* operon-I promoter given their shared architecture.

Both promoter regions have a CcpA binding site (*cre* box) overlapping the −35 box (Supplementary Fig. S18B and C), consistent with the observation that CcpA also controls these operons (Fig. 9A) [22]. CcpA knockout experiments demonstrate that both operons are repressed in the presence of glucose through binding of CcpA to the *cre* box [22]. Glucose-induced repression of the genetic machinery specific for alternative carbon sources such as sialic acid is a theme in bacteria, first discovered in the well-characterized *E. coli lac* operon [88, 89]. This phenomenon exists in Gram-positive bacteria as well [90, 91], and in *S. pneumoniae* CcpA controls global gene expression in response to glucose concentrations [29]. Here, *S. pneumoniae* prioritizes glucose uptake and metabolism when present, consistent with the first growth phase of the diauxic pattern seen in Fig. 2.

**Figure 9.**
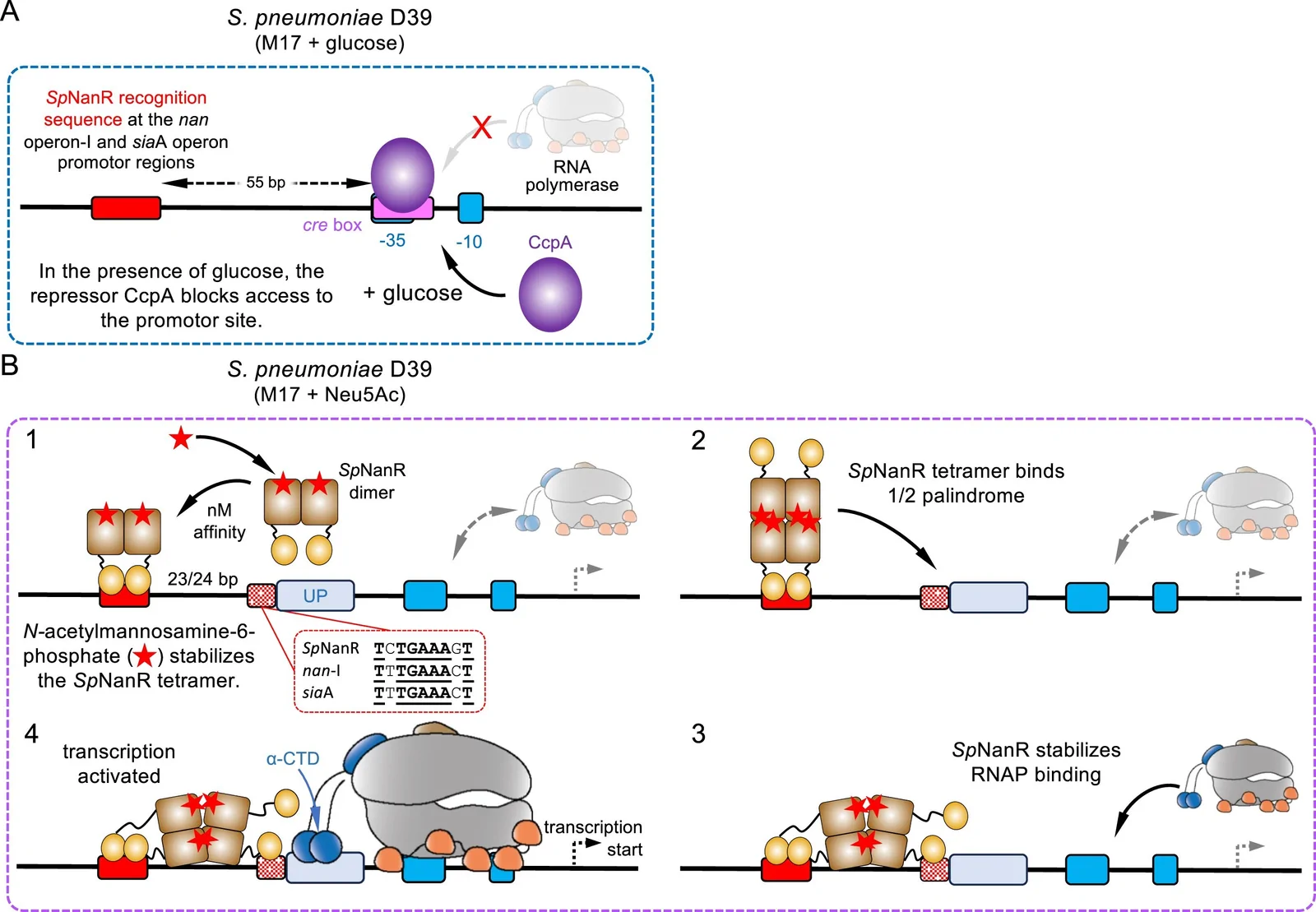
Proposed mechanisms for regulating the transcription of *nan* operons. (**A**) In response to glucose availability, the global regulator CcpA (purple oval) binds to the *cre* site (pink) that overlaps the −35 box (blue) and represses transcription of *nan* operon-I by preventing RNA polymerase from binding. (**B**) In the presence of Neu5Ac in the medium, expression of the *nan* operon-I and *sia*A operon is upregulated. To explain this phenotype, we propose that *Sp*NanR binds *N*-acetylmannosamine-6-phosphate (red star) and stabilizes the tetramer, which then binds a degenerate half-site 23 or 24 base pairs downstream of the *Sp*NanR recognition sequence (steps 1 and 2). The half palindrome sequences found in the *nan* operon-I and *sia*A operon are compared to the *Sp*NanR recognition sequence (inset). The half palindrome is adjacent to the α-CTD binding site (UP element, light blue) and the *Sp*NanR DNA-binding domain stabilize the α-CTD, promoting transcription (steps 3 and 4).

In contrast, the *nan* operon-II transcript is not upregulated when *S. pneumoniae* is grown on sialic acid-rich media [22]. We identified a possible *Sp*NanR recognition sequence site within the *nan* operon-II promoter region (Supplementary Fig. S18D), however it is the most divergent of the sequences (Fig. 1A) and includes a T to G mutation at position 10 that we demonstrate abolishes *Sp*NanR binding to the *nan*E recognition sequence (Fig. 7D, open red box) and therefore this site is likely to be nonfunctional. Also, this potential *Sp*NanR recognition sequence is downstream of the RNA polymerase −35 and −10 boxes, which suggests that if there is *Sp*NanR binding to this site it likely represses transcription. It is possible that *nan* operon-II, which encodes an ABC transporter, neuraminidase, and an oxidoreductase predicted to metabolize unusual sialic acids or other amino sugars, is regulated by an additional, as yet unidentified transcriptional regulator.

Across the *nan* gene cluster there are two further *Sp*NanR recognition sequences with potentially cryptic functions and we demonstrate that *Sp*NanR can bind to both sites with nanomolar affinity. The functional *Sp*NanR recognition sequence upstream of the *nan*A gene is within *nan* operon-I (Supplementary Fig. S18E), is not upregulated when *S. pneumoniae* is grown on sialic acid, and is considered nonfunctional [22]. This makes sense because it lies within the *nan* operon-I transcript, which is controlled by the promoter upstream of the *nan*E gene. However, the region has identifiable −35 and −10 boxes, suggesting it may be functional as a promoter for the final two genes in the operon, perhaps with the help of additional unknown regulators. The *sia*A operon also contains a *bona fide Sp*NanR recognition sequence located upstream of the operon, with pseudo −35 and −10 boxes orientated for transcription in the opposite direction (Supplementary Fig. S18F). However, there is no evidence that this region is transcribed, and the sequence contains early stop codons in all three reading frames. Overall, the functional significance of having *Sp*NanR recognition sequences located within *nan* operon-I upstream of the *nan*A gene and downstream of the *sia*A operon remains unclear.

### Trends and differences between SpNanR and other RpiR regulators

All RpiR regulators reported to date are repressors, whereas *Sp*NanR functions as a transcriptional activator. Moreover, effector binding to RpiR regulators is reported to modulate DNA binding, whereas our biochemical and structural data demonstrate that effector binding to *Sp*NanR does not modulate DNA binding and instead stabilizes a higher order tetrameric state. To varying degrees, the molecular basis for repression has been studied in three cases: *V. vulnificus* NanR (*Vv*NanR), which also controls sialic acid metabolism [41]; *E. coli* MurR (*Ec*MurR), which controls cell-wall recycling [36, 40]; and *P. aeruginosa* RccR (*Pa*RccR), a global regulator of carbon metabolism [42, 43]. Our structural and functional characterization of *Sp*NanR highlights conserved RpiR features as well as distinct differences.

Given the sequence similarity between these RpiR regulators, it is unsurprising that they share very similar DNA-binding and sugar isomerase domain structures. In all four cases (*Sp*NanR, *Vv*NanR, *Ec*MurR, and *Pa*RccR) the effector ligands are phosphorylated carbohydrates (*N*-acetylmannosamine-6-phosphate, *N*-acetylmuramate-6-phosphate, and 2-keto-3-deoxy-6-phosphogluconate, respectively) that bind to a site situated identically in their sugar isomerase domains. Moreover, the phosphate binding loop (i.e. loop-7 of *Sp*NanR) is highly conserved, comprising side chain and main chain contacts to the phosphate moiety. In contrast, the loops that bind the effector ligand distal from the phosphate are rearranged depending on the ligand the RpiR regulator binds. Nonetheless, because of the location of the effector binding site at the tetrameric interface, all effector ligands bridge residues from three monomers across the tetramer. For *Sp*NanR, at least, this provides a mechanism by which the effector ligand stabilizes the tetramer.

All crystal structures of full-length RpiR regulators or isolated sugar isomerase domains are tetrameric, including *Sp*NanR, since the protein concentration used during crystallization is very high (>>*K*_D_^4-2^). Tetramerization has been validated across these systems using solution-based approaches. For example, size exclusion chromatography and analytical ultracentrifugation experiments demonstrate that *Ec*MurR [36, 40] and *Pa*RccR [42, 92] are tetrameric, and this is generally considered the biologically relevant assembly. Although a *Vv*NanR dimer is proposed to bind DNA, multiple oligomeric states have been reported (dimer, tetramer, hexamer) by size exclusion chromatography [41, 93]. The effect of effector binding on oligomeric state has not been systematically investigated for these RpiR regulators. However, given the behavior of *Sp*NanR and the observation that the effector molecules bridge the tetramer, oligomerization may play a more significant role in RpiR function than previously appreciated.

The reported RpiR structures also highlight another important difference. In both *Vv*NanR and *Pa*RccR, the DNA-binding domains are tightly coupled to the sugar isomerase domain (Supplementary Fig. S12A and B), enabling direct communication between the two domains. Notably, the 16-residue linker in *Pa*RccR forms a short α-helix that positions the DNA-binding domains adjacent to the sugar isomerase domain [42, 43]. This arrangement, coupled with significant secondary structure within the homologous RpiR linker sequences, provides a mechanism for allosteric control of DNA binding in response to effector binding. In contrast, in-crystal and in-solution studies show the *Sp*NanR DNA-binding domains are extended away and are not engaged with the isomerase domain, irrespective of effector ligand or DNA binding (Supplementary Fig. S12C and D), providing a rationale for why *N*-acetylmannosamine-6-phosphate does not affect the affinity between *Sp*NanR and DNA. If the DNA-binding and sugar isomerase domains are independent,i.e. not coupled, there is no way to transmit a structural signal induced by effector binding between the two domains.

### How does *Sp*NanR sense sialic acid and upregulate transcription of nan operon-I?

Because the *nan* operon-I promoter region is responsive to sialic acid in the growth media and is best characterized by us (biophysically/structurally) and others (functionally), we consider how this locus is affected by the presence of an *Sp*NanR recognition sequence. Despite the *Sp*NanR recognition sequence being distal to the RNA polymerase −35 box of *nan* operon-I (55 base pairs) and the effector *N*-acetylmannosamine-6-phosphate not modulating DNA affinity, functional studies demonstrate that sialic acid significantly upregulates the *nan* operon-I transcript and that activation is abolished in a *nan*R knockout strain [22]. We demonstrate that *N*-acetylmannosamine-6-phosphate does, however, stabilize a tetrameric form. We consider two possibilities.

An obvious effect of having multiple binding sites distributed across the *nan* gene cluster is that, upon forming a tetramer, *Sp*NanR can bridge these sites. This interaction effectively loops the 2D linear arrangement of the cluster to form a 3D structure. This behavior has been established in other bacterial transcriptional regulators. AraC, for instance, forms DNA loops in its tetrameric form, repressing transcription until the effector arabinose binds, which disrupts the tetramer and the subsequent dimers promote RNA polymerase binding and transcription [94–98]. Unlike AraC, which dissociates into dimers upon effector binding and activates transcription, the effector *N*-acetylmannosamine-6-phosphate stabilizes the tetrameric form of *Sp*NanR, thereby enhancing its potential to loop DNA without directly altering its binding affinity. An advantage of DNA looping is that it leads to higher concentration of *Sp*NanR within the vicinity of the *nan* gene cluster, although how this specifically activates transcription of *nan* operon-I is unclear. Another possibility is that the formation of the loop affects the local DNA structure, enhancing the affinity of σ factors or auxiliary proteins for the site and thereby promoting RNA polymerase function.

Although *Ec*MurR and *Vv*NanR are repressors, where effector binding decreases their affinity for DNA, their binding mode and promoter architecture is intriguing. It is proposed that an *Ec*MurR tetramer bridges two adjacent DNA binding sites that overlap the polymerase binding sites (−35 and −10 boxes), explaining repression by *Ec*MurR. These sites are separated by 17 base pairs, and the tetramer would span a total 47 base pairs [36]. Interestingly, *Vv*NanR also binds two adjacent sites (NANRBI and NANRBII) each ∼55 base pairs in length [93]. In this case, *Vv*NanR dimers are proposed to bind to each site, although *Vv*NanR tetramers could plausibly bind [93]. The minimal DNA sequence that *Vv*NanR binds has not been defined, although the two regions span ∼107 base pairs.

Inspection of both the *nan* operon-I and the *sia*A operon promoter region does not identify adjacent *Sp*NanR binding sites, as found in the promoter regions that *Ec*MurR and *Vv*NanR bind, although there is a degenerate half palindrome ∼23 base pairs downstream of the *Sp*NanR sites in each operon (Fig. 9B). Despite being degenerate and unlikely to bind *Sp*NanR in isolation, the affinity would be increased through avidity—that is, the proximity of the *Sp*NanR binding site increases the local concentration of *Sp*NanR DNA-binding domains making even weak interactions more likely. Analysis of our *Sp*NanR structure suggests that the affinity of the DNA-binding domains for DNA is determined largely through backbone interactions (Fig. 7), suggesting even a degenerate half palindrome sequence could bind a *Sp*NanR DNA-binding domain, where specificity is driven by the bona fide sequence downstream. Given that this site is immediately adjacent to the RNA polymerase α-CTD binding site (UP element) in *nan* operon-I and the *sia*A operon (Supplementary Fig. S18B and C), one hypothesis is that *N*-acetylmannosamine-6-phosphate stabilizes the tetramer, which can bridge across the two sites with one DNA-binding domain engaging the half palindrome (Fig. 9B, steps 1 and 2). The distance *Sp*NanR would need to span across a linear DNA sequence is ∼150 Å, which is consistent with the distance of the extended tetramer in solution (Supplementary Table S9 and  Supplementary Fig. S19, ∼160 Å). The flexibility of the *Sp*NanR linker region (residues 77–88), as demonstrated by our structural studies, would provide the domains with significant flexibility to bind at both sites. The presence of a *Sp*NanR DNA-binding domain at the half palindrome could enhance RNA polymerase binding through direct interactions with the α-CTD and stabilize binding (Fig. 9B, steps 3 and 4). In *E. coli*, binding of the RNA polymerase α-CTD to sequences upstream of the −35 box dramatically affects transcription rates [99, 100]. This hypothesis is consistent with our structural and biophysical data, and the previously reported activation of *nan* operon-I and the *sia*A operon in sialic acid containing media. A half-palindromic sequence is absent from the *nan*A promoter region (Supplementary Fig. S18E), consistent with the observation that this region is not activated by *Sp*NanR. It is also, to date, a unique mechanism for an RpiR regulator.

This model is also consistent with *Sp*NanR knockout studies, which result in significant decreases in expression of the *nan* operon-I transcript in M17 media with or without glucose or sialic acid supplementation (see fig. 2A and B in their work [22]), meaning that simply binding *Sp*NanR to the *nan* operon-I promoter region induces some expression. We demonstrate that *Sp*NanR binds to its recognition sequence in the *nan* operon-I promoter region with nanomolar affinity even in the absence of its effector *N*-acetylmannosamine-6-phosphate (Fig. 5). Although the dissociation constant for *Sp*NanR in the absence of *N*-acetylmannosamine-6-phosphate is weak (*K*_D_^4-2^ = 97 μM), a tetramer can form and would be expected to reside intermittently at the *Sp*NanR recognition sequence. Again, the tetrameric *Sp*NanR could bridge across and one of the DNA-binding domains binds the half palindrome. Here, avidity could play a role in stabilizing and promoting complex formation. From a biological perspective, this means that *Sp*NanR stimulates baseline transcription of the *nan* operon-I. The low-level expression of the sialic acid machinery, including a sialic acid ABC transporter, presumably allows *S. pneumoniae* to sense the presence of sialic acid in the environment.

## Conclusions

Our study reveals that *S. pneumoniae* NanR is a unique case within the RpiR family, functioning as a transcriptional activator rather than a repressor. Structural and biochemical analyses demonstrate that the effector, *N*-acetylmannosamine-6-phosphate, does not alter DNA-binding affinity but instead stabilizes tetramer formation, establishing a ligand-dependent oligomerization mechanism that is distinct from previously characterized RpiR proteins. This decoupling of effector sensing from direct modulation of DNA binding expands the known repertoire of bacterial transcriptional regulation strategies. These findings provide a molecular framework for understanding sialic acid-responsive gene activation in pneumococci, and future work should aim to validate these mechanisms *in vivo*.

## Supplementary Material

gkag953_Supplemental_Files

## Data Availability

The data that support this study are available from the corresponding author upon reasonable request. The atomic models for *Sp*NanR (PDB ID: 8V5F), SpNanR with *N*-acetylmannosamine-6-phosphate (core tetramer) (PDB ID: 8TX9), *Sp*NanR with *N*-acetylmannosamine-6-phosphate (DNA-binding domains) (PDB ID: 8V4V), and *Sp*NanR with the nanE DNA sequence (PDB ID: 9DQN) are available through the Protein Data Bank at https://doi.org/10.2210/pdb8V5F/pdb, https://doi.org/10.2210/pdb8TX9/pdb, https://doi.org/10.2210/pdb8V4V/pdb, and https://doi.org/10.2210/pdb9DQN/pdb, respectively. The nanR gene and protein sequence is available through the UniProt database with the accession code A0A0H2ZPE9.
