# Supplementary material for: Mechanism of NanR transcriptional activation of sialic acid metabolism in Streptococcus pneumoniae
Source: Nucleic Acids Res. 2026 Oct 5;54(18):gkag953. doi: 10.1093/nar/gkag953 (PMC13635753; doi:10.1093/nar/gkag953)
Supplement: Supplementary file 1 [file gkag953_supplemental_files.zip › Supplementary Movie 1 Figure Legend.docx]

**Supplementary Movie 1 | Comparison of canonical B-DNA structure and the DNA distortion in the *Sp*NanR – DNA crystal structure.** Morph of the DNA structure in the SpNanR – DNA crystal structure and typical B-DNA sequence demonstrates the distortion of DNA upon *Sp*NanR engagement.
